# Integrated immunodominant epitope discovery for dual-purpose rapid and economical diagnostic and immunoprotective applications against MRSA

**DOI:** 10.3389/fimmu.2025.1697829

**Published:** 2025-10-20

**Authors:** Longlong Chen, Pengju Yan, LianLi Duan, Guangyang Ming, Xiaoqiong Wang, Jinyong Zhang, Zhifu Chen, Qiang Gou, Yue Yuan, Haiming Jing, Ping Cheng, Ping Luo, Hao Zeng, Zhiyong Liu, Quanming Zou, Zhuo Zhao

**Affiliations:** ^1^ National Engineering Research Center of Immunological Products, Department of Microbiology and Biochemical Pharmacy, College of Pharmacy and Laboratory Medicine, Army Medical University, Chongqing, China; ^2^ Department of Laboratory Medicine, Southwest Hospital, Army Medical University, Chongqing, China

**Keywords:** *Staphylococcus aureus*, immunodominant epitope, lethal sepsis, diagnostic, vaccine

## Abstract

Current diagnostic and preventive strategies against *Staphylococcus aureus* methicillin-resistant strains (MRSA) remain inadequate. Hence, we aimed to identify candidate epitopes as potential therapeutic targets and diagnostic biomarkers. We focused on clinically validated targets and investigated four antigens (Hla, SEB, MntC, and IsdB) currently incorporated into phase III clinical trials of a recombinant five-antigen vaccine (termed rFSAV) and the recently identified leukocidin LukG. Using convalescent serum samples from patients with clinically confirmed MRSA, we identified 10 immunodominant epitopes through ELISA screening of overlapping 18-mer peptides, seven of which named MntC_55-72_, MntC_121-138_, MntC_271-285_, SEB_37-54_, LukG_30-47_, LukG_235-252_, and LukG_246–263_ have not been previously reported. Immunoprotection trials showed that five epitopes Hla_168–185_, IsdB_384–401_, MntC_55–72_, SEB_37–54_, and LukG_235–252_ elicited effective protection in a BALB/c murine sepsis model infected with MRSA252. The combination of these protective epitopes exhibited broad-spectrum efficacy against both the MRSA252 strain and phylogenetically distinct clinical isolates. Diagnostically, the performance of the epitope panel was superior to that of conventional culture methods with a sensitivity of 0.839 and specificity of 0.826 in a 3-h detection window, thus offering rapid and cost-effective advantages. Notably, bioinformatic analysis showed that all identified B-cell epitopes contained predicted CD4^+^ T-cell epitope sequences, which suggests the potential to elicit combined T–B cell immune responses through MHC-II presentation. Thus, these immunodominant epitopes with dual functions that integrate both diagnostic and immunoprotective capabilities could function as a novel immunodiagnostic toolkit that enables rapid MRSA detection and aid in establishing a multi-epitope vaccine platform. These findings present an integrated strategy that bridges diagnostic development and vaccine design for MRSA management.

## 1 Introduction

*Staphylococcus aureus* infections account for a disproportionately high number of fatalities worldwide (1). It causes a wide variety of diseases including skin and soft tissue infections, endocarditis, osteomyelitis, bacteremia, and fatal pneumonia (2). A major cause of *S. aureus* devastation is antibiotic resistance, and methicillin-resistant *Staphylococcus aureus* (MRSA) bloodstream infections show the highest mortality rate attributable to common gram-positive multidrug-resistant bacteria in intensive care units and second highest mortality rate among all drug-resistant bacteria (3). The escalating consumption of healthcare resources has imposed a cumulative economic burden and created critical biosecurity vulnerabilities in the United States (4), China (5), United Kingdom (6), Japan (7), and other countries. Current clinical bacterial diagnostic methods such as bacterial culture are often complex and time-consuming. Therefore, innovative diagnostic and nonantibiotic immunization approaches are urgently required for the clinical diagnosis and prevention of MRSA infections. Currently, few practical diagnostic methods have been developed (8), and vaccine candidates in development are either in the preclinical or early clinical stages with some failing to elicit protection in human subjects (9). The development of rapid diagnostic modalities for MRSA infection is a critical determinant of clinical outcomes, particularly for sepsis management (10). Early pathogen identification enables the timely initiation of targeted antimicrobial therapy, thereby reducing the critical window between symptom onset and appropriate antibiotic administration. Thus, the development of rapid diagnostic methods would substantially improve diagnostic efficiency and shorten the time required for drug treatment decisions, which would significantly increase patient survival rate (11), especially in sepsis cases.

*Staphylococcus aureus* infection requires the production of surface proteins for bacterial adhesion to host tissues, secretion of extracellular toxins and enzymes for destruction of host cells and tissues, and evasion or inactivation of the host immune system (12). Hence, diagnostic methods and vaccine-based immunoprotection must simultaneously target several factors with different effects to achieve comprehensive diagnostic value and immunoprotection. rFSAV is a recombinant penta-antigen *S. aureus* vaccine developed in our laboratory (13). Phase II clinical trials (CTR20181788, http://www.chinadrugtrials.org.cn/) have verified its immunogenicity and defense against *S. aureus* infections. Currently, phase III clinical trials (CTR20221329, http://www.chinadrugtrials.org.cn/) are underway. Alpha hemolysin (Hla), enterotoxin B (SEB), manganese transporter C (MntC), and iron-regulated surface determinant protein B (IsdB) are four antigens within rFSAV. While active immunization with Hla (14), SEB (15), MntC (16), and IsdB (17) individually provided partial protection against *S. aureus*, passive immunization utilizing monoclonal antibodies targeting these antigens afforded protection in a mouse sepsis model (18). Additionally, bicomponent leukocidins are a key factor for the immune evasion of *S. aureus*. Immunity targeting leukocidins blocks their cytotoxic and immunosuppressive effects *in vivo*, thereby conferring protection against bloodstream infections (19). *Staphylococcus aureus* produces five different leukocidins: gamma-hemolysin (HlgAB, HlgCB), leukocidin PVL (LukSF), LukED, and LukGH (also known as LukAB) (20). LukGH expression is significantly elevated compared to that of the other leukocidal cytokines after phagocytosis of *S. aureus* by neutrophils and is key to neutrophil evasion by *S. aureus* (21). Furthermore, LukG and LukH are secreted as monomers, which regulate the expression of inflammatory cytokines in neutrophils in their monomeric form (22). Thus, immunization using the monomeric form of LukG may elicit critical immunoprotection. Moreover, the use of these five antigens for diagnosis could maximize the accuracy of diagnostic tests.

The production of specific antibodies during the humoral immune response plays a vital protective role against MRSA infections (23). Antibody-based therapeutics are emerging as promising strategies for combating drug-resistant bacterial infections (24). Current vaccine development strategies show that whole-antigen vaccines exhibit inferior efficacy compared with those of epitope-specific formulations (25) as protective immunity can be effectively elicited even by limited immunodominant epitopes (26). Immunodominant epitopes have been systematically characterized across phylogenetically diverse bacterial pathogens (27–29). Consequently, identifying the immunodominant epitopes within these five antigens that drive protective B-cell responses in infected populations is critically important. Furthermore, these immunodominant epitopes may be easily identified in the antigens based on the strong immune response that they induce (30). Antibodies developed during the convalescent phase of infection show significant protective effects against pathogenic reinfection and act as critical components of adaptive immunity (31). As Hla, SEB, MntC, IsdB, and LukG are MRSA autoantigens that are recognized by autoantibodies in the sera of MRSA-infected patients, population-specific immunodominant epitopes have been successfully identified using sera from infected individuals (32). The immunodominant epitopes thus identified are potential diagnostic markers and immunoprotective targets and play an improved role in diagnosis and protection.

In this study, we used the clinical MRSA-infected population as the model to systematically identify immunodominant B-cell epitopes in the five antigens through the immunological analysis of convalescent sera from patients with clinical MRSA. Four epitopes were derived from the four distinct rFSAV components Hla, SEB, MntC, and IsdB and one epitope from the LukG toxin. Experiments were performed in BALB/c murine infection models to validate the protective immunity elicited by these epitopes against MRSA252 strains. Furthermore, epitope cocktail formulations were used to identify synergistic protection against heterologous clinical isolates. Further serological assays were performed to determine the diagnostic sensitivity and specificity of the candidate epitopes in detecting serum IgG in patients with active infections, which would indicate their dual potential as therapeutic targets and diagnostic biomarkers.

## 2 Materials and methods

### 2.1 Ethics statement

All animal and human experiments were approved by the Laboratory Animal Welfare and Ethics Committee of the Army Medical University (Chongqing, Permit No. AMUWEC2019027). The experiments were performed in accordance with the approved guidelines. We obtained informed consent from all participants.

### 2.2 Animals, antigens, and antiserum

BALB/c mice (6–8-week-old, specific pathogen-free, female) were purchased from Sichuan Weitong Lihua Experimental Animal Technology Co., Ltd. (Sichuan, China). Peptide–keyhole limpet hemocyanin (KLH) conjugate was performed for each immunodominant peptide by GL Biochem Ltd. (Shanghai, China). A total of 30 convalescent serum samples were collected from MRSA-infected patients at Southwest Hospital, Army Medical University (Chongqing, China), including 16 from bacteremia cases, and 14 from localized infection cases (9 with skin and soft tissue infection, 5 with pneumonia). MRSA infection was confirmed in all patients through bacterial culture and clinical drug susceptibility testing. Antisera with titers > 1:6400 were selected for subsequent epitope mapping.

The MRSA252 strain was acquired from the American Type Culture Collection (ATCC; Manassas, VA, USA). Bacterial stocks were cultured on Mueller–Hinton agar (MHA) plates at 37°C, and a single colony was inoculated into Mueller–Hinton broth (MHB) for overnight growth. Next, 100 μL were transferred into 10 mL of fresh MHB and cultured for approximately 4 h. The supernatant was removed through centrifugation and diluted with phosphate-buffered saline (PBS) to obtain the desired colony concentration.

### 2.3 Linear B-cell epitope mapping

Next, 18-mer peptides with 12 amino acid length overlaps to cover the full lengths of Hla (Sequence ID: ADQ77533.1), IsdB-N2 (Sequence ID: WP_031875332.1), MntC (Sequence ID: WP_095231761.1), SEB (Sequence ID: AUT32286.1), and LukG (Sequence ID: WP_000595324.1) were synthesized and purified by GL Biochem Ltd. (Shanghai, China). Additionally, OVA_192–201_ (EDTQAMPFRV) synthesized by the same company and bovine serum albumin (BSA) (Sequence ID: NP_851335.1) served as negative control peptide and protein, respectively. The peptides (purity ≥ 95%) were dissolved in dimethyl sulfoxide (1 mg/mL) and stored at −80°C.

The serum samples were diluted 1:300 (v/v) in PBS. Nonspecific binding was prevented by blocking the coated microtiter plates with PBS (pH 7.4) containing 2% BSA. Peroxidase-conjugated goat anti-human IgG antibodies (Solarbio, Beijing, China) at 1:3500 dilution were used as the secondary antibodies. The ELISA results are expressed as absorbance at 450 nm. The normal value for each peptide was calculated by testing the sera from healthy humans. The positive threshold was defined as 2.1-fold above the mean absorbance of negative control serum.

### 2.4 Immunization and infection

To determine the protective efficacies of Hla, SEB, MntC, IsdB, and LukG immunodominant peptides, the mice were randomized into different groups and intramuscularly injected with 100 μg of an individual immunodominant peptide–KLH conjugate + Quil-A adjuvant (n = 8), or Hla, SEB, and LukG immunodominant peptide–KLH mixture (Mix3) (n = 10), or Hla, SEB, MntC, IsdB, and LukG immunodominant peptide–KLH mixture (Mix5) (n = 10), or Quil-A adjuvant + PBS (n = 8 or 10), or PBS alone on days 0, 14, and 21 (n = 8 or 10). Then, 1 week after the last booster, mice in the Mix3, Mix5, Quil-A + PBS, and PBS alone groups were infected with 6×10^8^ or 8×10^7^ CFU/mL of *S. aureus* strain MRSA252 in 100 μL saline via tail vein injection. The survival rate in each group was monitored for 7 days.

### 2.5 Bacterial burden, severity score, and tissue histology

Seven days post-immunization (n = 8/group), mice were challenged with 8×10⁷ CFU MRSA252. Kidneys and lungs were aseptically harvested 48 h post-infection, homogenized in PBS, and serially diluted (5-fold). Homogenates were plated on MHA and incubated 24 h at 37°C. Bacterial burden was expressed as CFU/organ.

The health status of the mice was assessed following MRSA252 sublethal infection by establishing a 0–4 scoring system based on their overall condition and fur status at 2 days post-infection. The scores were assigned as follows: 0, healthy mice with smooth and glossy fur and no abnormalities; 1, mildly lethargic appearance with reduced activity but normal feeding and slight fur with mild ruffling; 2, significant lethargy, reduced activity and feeding, mild fever or weight loss, and ruffled fur with mild hair loss; 3, extremely lethargic, no feeding, fever or significant weight loss, respiratory distress, cyanosis, severely ruffled fur, significant hair loss, and skin redness or inflammation; and 4, severe infection symptoms such as shock or coma, severe secondary organ damage, extensively ruffled fur, extensive hair loss, and skin ulcers or infections.

For histopathological analysis, organs were fixed in 4% paraformaldehyde, paraffin-embedded, sectioned at 4 μm, and stained with hematoxylin and eosin for microscopic evaluation.

### 2.6 Structural localization and sequence alignment of immunodominant epitopes

The immunodominant peptides were mapped onto the Hla, SEB, MntC, IsdB, and LukG 3D structures (PubMed protein database) using PyMOL 1.1. Hla, SEB, MntC, IsdB, and LukG sequences from different *S. aureus* strains were retrieved from the GenBank database for alignment using the National Center for Biotechnology Information Basic Local Alignment Search Tool (BLAST).

### 2.7 B cell, Th cell, and CTL epitope prediction

The CTL epitopes of the five proteins were predicted using SYFPEITHI and NetCTL. The Th cell epitopes of the five proteins were predicted using SYFPEITHI and NetMHCIIpan 4.0. B cell epitopes were predicted using the Protean module of DNASTAR Lasergene.

### 2.8 Challenge with clinical MSRA isolates

Challenge strains were selected from distinct phylogenetic clades based on evolutionary analyses reported previously (33). Three geographically diverse Chinese MRSA isolates (CQ19, BJ2, GZ9) were used. Mice received intravenous challenges of 4×10⁷ CFU per strain via tail vein, with concurrent administration of either MIX3 + Quil-A (test group) or Quil-A + PBS (control group). Survival analysis was performed using the Kaplan-Meier estimator.

### 2.9 Diagnostic experiments

The identified epitopes were used to develop diagnostic assays. The five immunodominance epitopes were coated on 96-well plates at a dose of 20 μg per well, and the sera of patients with clinical MRSA infection and those of uninfected individuals were selected for detection. The dilution was 1:300, and sheep anti-human IgG was used as the secondary antibody at a dilution of 1:5000. The sensitivity and specificity of these assays were evaluated using a panel of clinical MRSA-infected and non-infected serum samples collected from Southwest Hospital. Among these, MRSA-positive samples (n = 31) were confirmed by clinical bacterial culture and drug sensitivity testing. Non-infected serum samples (n = 24) comprised two well-characterized non-MRSA groups confirmed by clinical drug sensitivity testing: 16 healthy controls with no history of MRSA infection or hospitalization and negative for *S. aureus* colonization (via nasal swab culture), and 8 disease controls with infection from non-MRSA pathogens (4 methicillin-sensitive *S. aureus*, 4 *Klebsiella pneumoniae*) verified by bacterial culture to exclude MRSA.

### 2.10 Statistical analysis

Statistical analyses were performed using GraphPad Prism 8.0. Data are expressed as the mean ± SD and were analyzed by one-way ANOVA with Bonferroni’s *post hoc* test. Significance was assigned at p < 0.05. Statistical analysis of the diagnostic test was performed using SPSS (version 19.0). McNemar’s paired χ² test was used for the comparison of two sample rates; the calibrated McNemar’s paired χ² test and exact probability method were used when necessary, and the Kappa test was used to measure the coincidence. The ELISA cutoff of the diagnostic test was determined using Youden’s J index method.

## 3 Results

### 3.1 Identification of immunodominant epitopes on Hla, SEB, MntC, IsdB, and LukG using sera from clinical patients infected with MRSA

**Figure 1:**
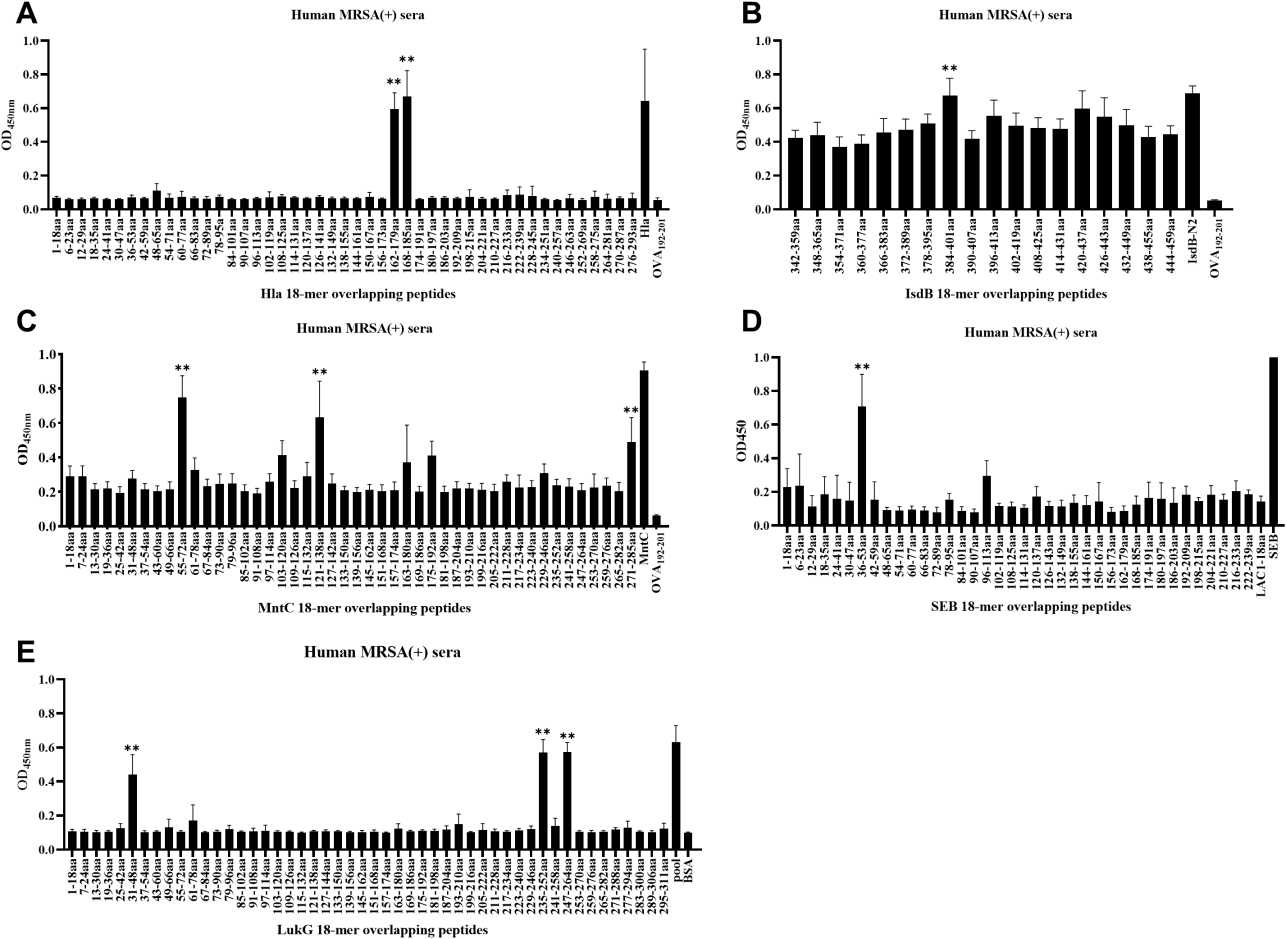
B-cell epitope mapping in Hla, SEB, MntC, IsdB, and LukG using ELISA results. To identify immunodominant epitopes of Hla **(A)**, IsdB **(B)**, MntC **(C)**, SEB **(D)**, and LukG **(E)**, microtiter plates were coated with synthetic overlapping peptides spanning each full-length antigen. Positive controls included full-length Hla, SEB, MntC, and IsdB proteins or the LukG peptide pool; negative controls used OVA_192–201_ or BSA. Sera from clinical MRSA patients served as primary antibodies. Absorbance was measured at 450 nm, with optical density (OD) values representing triplicate experiment averages from all nine patients per group. Data are represented as the means ± SD. Significant differences are indicated as **p < 0.01.

Linear B-cell epitope mapping was performed by ELISA screening of overlapping 18-mer peptides against convalescent sera from MRSA patients. As shown in **Figure 1**, we identified 10 distinct epitopes (Hla_162–179_, Hla_168–185_, IsdB_384–401_, MntC_55–72_, MntC_121–138_, MntC_271–285_, SEB_37–54_, LukG_30–47_, LukG_235–252_, and LukG_246–263_) with strong IgG reactivity. The epitope sequences are listed in **Table 1**. The immunodominant epitopes Hla_162–179_, Hla_168–185_, and IsdB_384–401_ may share the same B-cell epitope as Hla_191–208_, Hla_194–211_, Hla_197–214_, and IsdB_384–401,_ which were previously identified using serum from antisera obtained from c-di-AMP + HI antisera (34) and sera from volunteers in a Phase 1b clinical trial of rFSAV (35). The other seven epitopes have not been previously reported and may harbor novel linear B-cell epitopes. Notably, systematic bioinformatic screening of candidate epitopes showed no detectable homology to established toxigenic domains in the virulence factor database.

**Table 1:** Sequence of the immunodominant epitopes on Hla, SEB, MntC, IsdB and LukG identified in this study.

The immunodominant epitopes	Sequence of the immunodominant epitopes
Hla_162-179_	DKKVGWKVIFNNMVNQNW
Hla_168-185_	KVIFNNMVNQNWGPYDRD
IsdB_384-401_	VMETTNDDYWKDFMVEGQ
MntC_55-72_	LTDADVILYNGLNLETGN
MntC_121-138_	LDNGIKYVKTIQQTFDNG
MntC_271-285_	KMMKSNIETVHGSMK
SEB_37-54_	INVKSIDQFRYFDLIYSI
LukG_31-48_	QKNITOSLOFNFLTEPNY
LukG_235-252_	MSHDKKDKGKS OFVVHYK
LukG_247-264_	FVVHYKRSMDFFKIDWNR

### 3.2 Immunization with individual immunodominant epitopes elicited different protective efficacies against MRSA252 challenge

To determine the protective role of individual immunodominant epitopes against MRSA infection, BALB/c mice were immunized with KLH–conjugated epitopes + Quil-A adjuvants, Quil-A + PBS, or PBS alone prior to MRSA252 infection. As MntC is an *S. aureus* membrane protein, we exclusively used the epitope peptide that showed the highest OD value during epitope screening in the immunoprotection trial. Owing to the inherent limitation in immunogenicity elicited by single epitopes, which may be inferior to the multi-epitope synergy of full-length proteins, we implemented a focused experimental design using murine bacterial colonization models to evaluate epitope-specific protective efficacy.

Considering the crucial role of adjuvants in enhancing immunogenicity of vaccines involving non-replicating, inactivated, and subunit antigens (36) and the insufficiency of antibodies alone in producing a response to various *S. aureus* strain-induced diseases, we designed a next-generation vaccine to stimulate a cellular immune response (37). We used Quil-A adjuvant, which induces both humoral and cellular immune responses (38), to compensate for the deficiency of B cell epitope immunity during a cellular response. In the MRSA sepsis model, systemic bacterial dissemination led to characteristic multi-organ colonization patterns. The kidneys and lungs are the commonly colonized organs (2).

**Figure 2:**
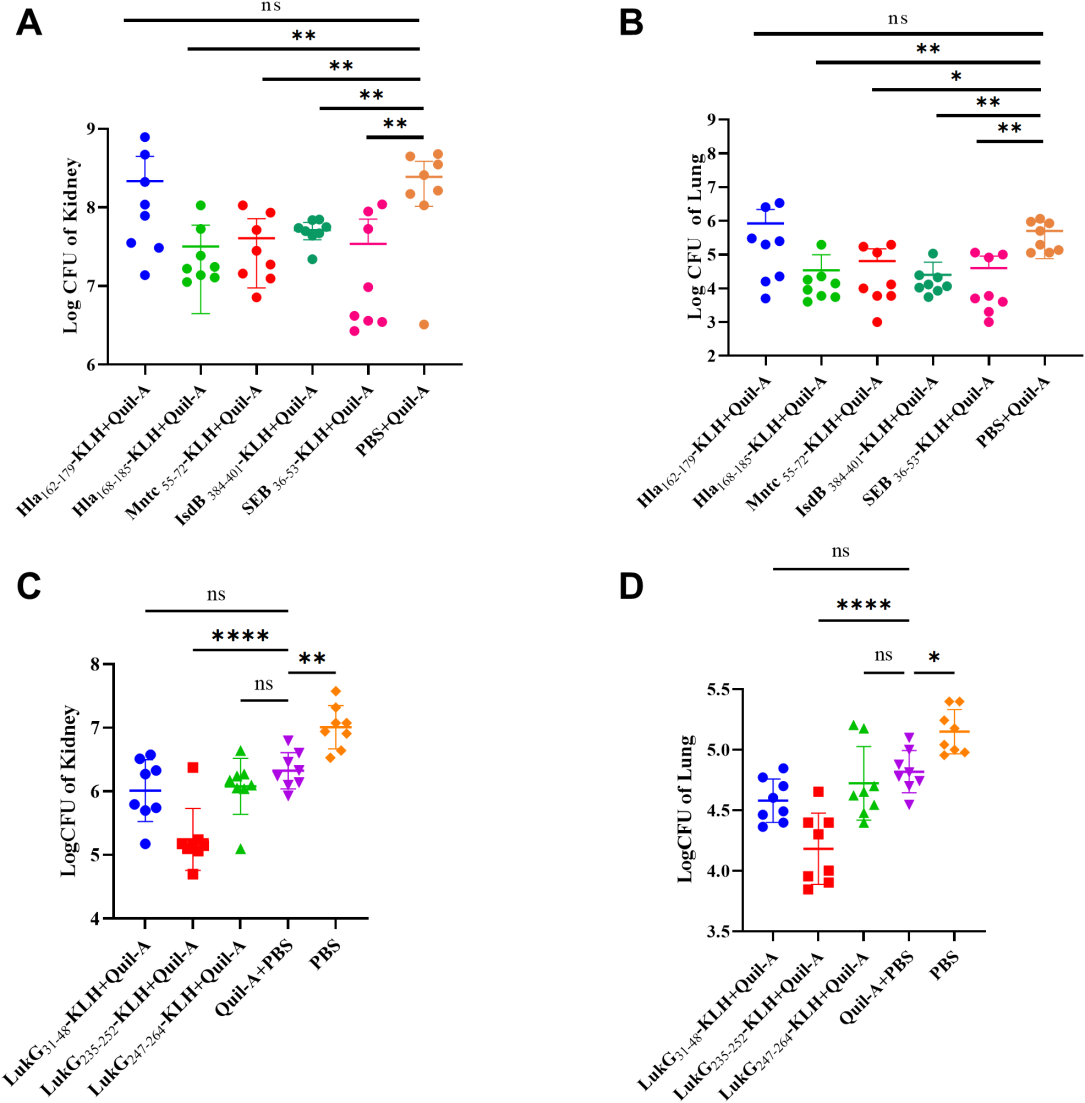
Bacterial burden in the lungs and kidneys of mice immunized with immunodominant epitope peptides after challenge with MRSA252 via tail vein injection. **(A)** Kidney bacterial burden in MRSA252-challenged mice immunized with Hla_168–185_, IsdB_384–401_, MntC_55–72_, SEB_37–54_-KLH + Quil-A, Quil-A + PBS, or PBS alone. **(B)** Lung bacterial burden in MRSA252-challenged mice immunized as described in **(A)**. **(C)** Kidney bacterial burden in MRSA252-challenged mice immunized with LukG_30–47_, LukG_235–252_, LukG_252–269_-KLH + Quil-A, Quil-A + PBS, or PBS alone. **(D)** Lung bacterial burden in MRSA252-challenged mice immunized as described in **(C)**. Significant differences are indicated as *p < 0.05, **p < 0.01, ****p < 0.0001. "ns" denotes "not significant". (n = 8 per group; two independent experiments).

The bacterial load was evaluated in the organs of the immunization group mice 48 h after MRSA252 challenge. The bacterial burden in the kidneys and lungs was significantly lower in mice immunized with Hla_168–185_-, IsdB_384–401_-, MntC_55–72_-, SEB_37–54_-, and LukG_235–252_-KLH + Quil-A than in those treated with Quil-A + PBS and PBS alone. Compared with Quil-A + PBS group: in the kidney, Hla_168–185_-KLH + Quil-A (p = 0.0036), MntC_55–72_-KLH + Quil-A (p = 0.0049), IsdB_384–401_-KLH + Quil-A (p = 0.0049), SEB_37–54_-KLH + Quil-A (p = 0.0043), and LukG_235–252_-KLH + Quil-A (p < 0.0001) (**Figures 2A, C**); in the lung, Hla_168–185_-KLH + Quil-A (p = 0.0084), MntC_55–72_-KLH + Quil-A (p = 0.0130), IsdB_384–401_-KLH + Quil-A (p = 0.0071), SEB_37–54_-KLH + Quil-A (p = 0.0087), and LukG_235–252_-KLH + Quil-A (p < 0.0001) (**Figures 2B, D**). The other immunodominant epitope peptides did not exhibit significant differences. Therefore, the effects of Hla_168–185_-KLH, IsdB_384–401_-KLH, MntC_55–72_-KLH, SEB_37–54_-KLH, and LukG_235–252_-KLH were stronger than those of the other epitopes and Quil-A + PBS.

### 3.3 Immunization with mix-epitope-KLH reduced MRSA252 infection in a lethal sepsis model

As the efficacy of a single immunodominant peptide is limited, we investigated whether these immunodominant peptides elicited an additive effect. BALB/c mice were immunized with the following cocktails: Hla_168–185_-KLH, MntC_55–72_-KLH, IsdB_384–401_-KLH, SEB_36–53_-KLH, and LukG_235–252_-KLH (Mix5-epitope-KLH); Hla_168–185_-KLH, SEB_36–53_-KLH, and LukG_235–252_-KLH (Mix3-epitope-KLH); and PBS + Quil-A or PBS alone prior to lethal MRSA252 challenge.

**Figure 3:**
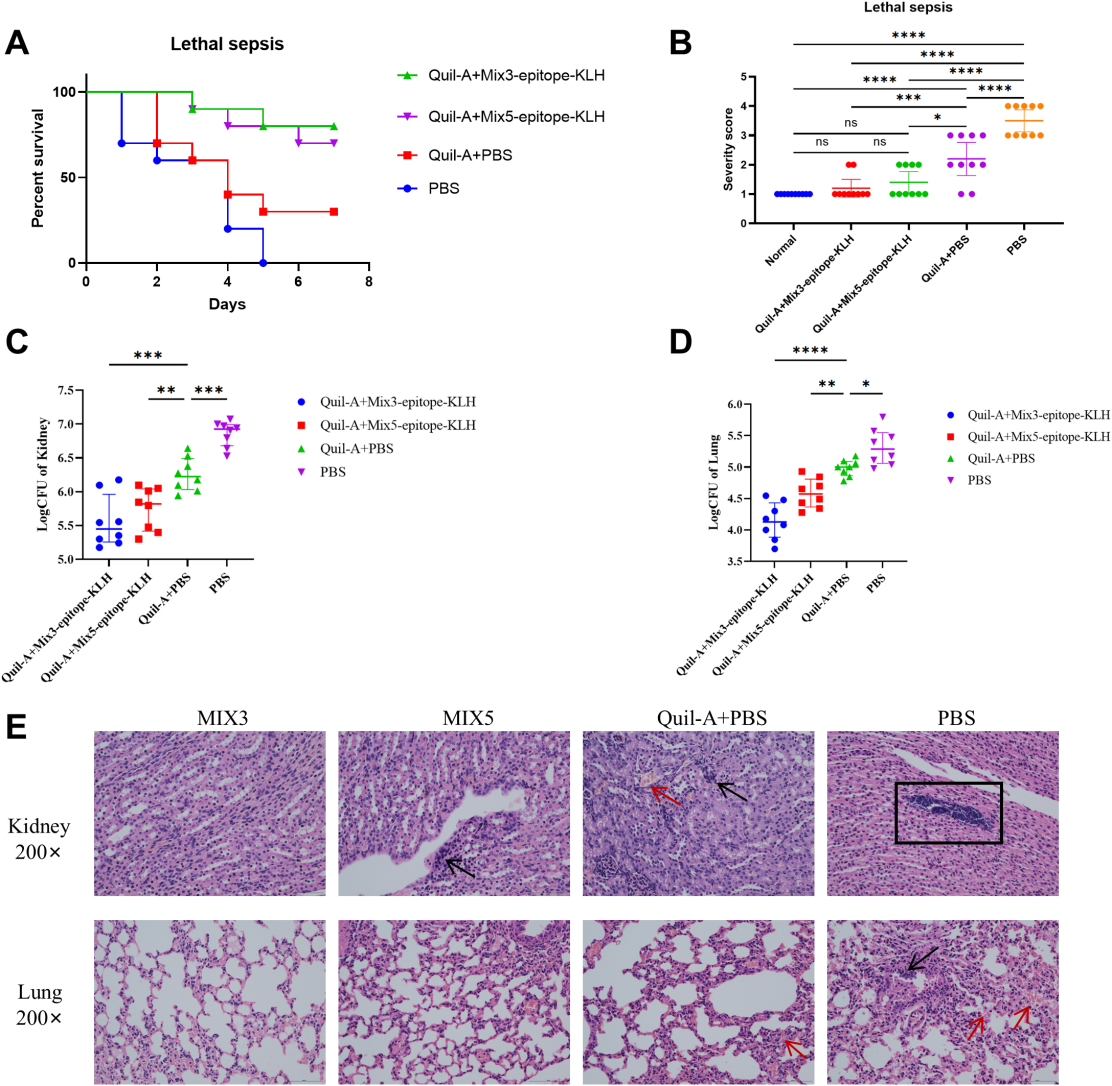
Multiple epitope vaccine-induced protective response against MRSA252 infection in immunized mice. **(A)** Percent survival against MRSA252 infection in immunized mice (n = 10 per group). **(B)** Severity scores of mice in the control group and other groups 48 h after challenge. *p < 0.05, ***p < 0.001, ****p < 0.0001 "ns" means p > 0.05.. (n = 10 per group) **(C, D)** Bacterial burden in the kidneys and lungs of mice after challenge with MRSA 252. *p < 0.05, **p < 0.01, ***p < 0.001, ****p < 0.0001. (n = 10 per group). **(E)** Histological analysis of MRSA challenged mice. Hematoxylin and eosin staining of kidney and lung sections at 48 h after sublethal infection. Microscopic images of kidneys 200× (top row) and lungs 200× (bottom row). The black arrows indicate inflammatory cell infiltration and inflammatory exudate. The rectangular box indicates abscess. The red arrows indicate bleeding. MIX3, Hla_168–185-_KLH, SEB_37–54-_KLH, and LukG_235–252-_KLH; MIX5, Hla_168–185-_KLH, SEB_37–54-_KLH, LukG_235–252-_KLH, IsdB_384–401-_KLH, and MntC_55–72-_KLH.

Survival analysis showed that 80% of the mice immunized with Mix3-epitope-KLH + Quil-A and 70% of the mice immunized with Mix5-epitope-KLH + Quil-A survived the MRSA252 challenge. These rates were significantly higher than that of mice immunized with PBS + Quil-A or PBS alone. Thus Mix-epitope-KLH immunization exerted a higher protective efficacy and exhibited an additive effect in controlling MRSA252 infection. The significance of the protective effect of Mix-epitope-KLH + Quil-A was evaluated using the log-rank (Mantel-Cox) test. Compared with the PBS + Quil-A group, the Mix3-epitope-KLH + Quil-A (p = 0.0199) and Mix5-epitope-KLH + Quil-A (p = 0.0510) groups had significantly higher protective efficacies. Similarly, the Mix3-epitope-KLH + Quil-A (p = 0.0002) and Mix5-epitope-KLH + Quil-A (p = 0.0004) groups had higher protective efficacies than the PBS alone group. No statistically significant difference was observed between the PBS + Quil-A and PBS alone groups (p = 0.1620) (**Figure 3A**).

At 48 h post-MRSA252 challenge, bacterial burden was significantly reduced in kidneys and lungs of Mix3- and Mix5-immunized mice. Thus, in the kidney, Mix3-epitope-KLH + Quil-A (p<0.0001) and Mix5-epitope-KLH + Quil-A (p=0.0046) reduced bacterial burden compared to that in the Quil-A + PBS controls (**Figure 3C**); in the lungs, Mix3-epitope-KLH + Quil-A (p<0.0001) and Mix5-epitope-KLH + Quil-A (p=0.0080) reduced bacterial burden compared to that in the Quil-A + PBS group (**Figure 3D**). Additionally, we scored the degree of severity according to the mouse health status (**Figure 3B**).

Histological analysis revealed preserved renal tubules and alveolar structures in MRSA252-challenged mice immunized with Mix3-epitope-KLH + Quil-A. In contrast, kidneys and lungs from Mix5-epitope-KLH + Quil-A-, PBS + Quil-A-, and PBS-immunized groups exhibited abscesses, bacterial colonies, and hemorrhagic lesions (**Figure 3E**). These findings demonstrate that Mix3-epitope-KLH + Quil-A conferred substantial protection against a MRSA252 challenge in the lethal sepsis model.

### 3.4 Localization and sequence alignment of immunodominant epitopes on Hla, SEB, MntC, IsdB, and LukG

**Figure 4:**
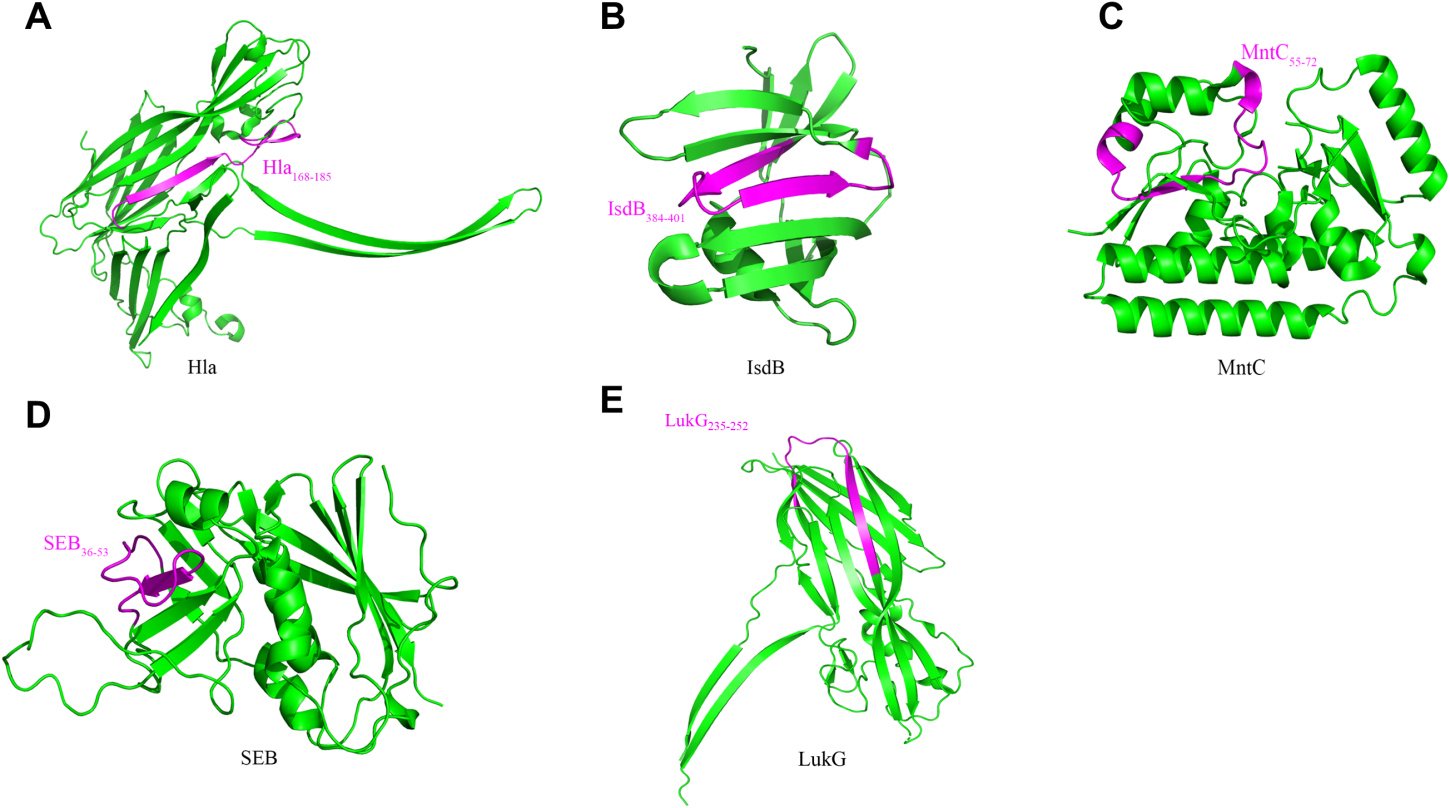
Localization of immunodominant epitopes on Hla, SEB, MntC, IsdB and LukG. The crystal structures of Hla (3anz.pdb), SEB (5xz0.pdb), LukG (6rhw.pdb), MntC (4nnp.pdb) and IsdB (3rtl.pdb) were obtained from PDB. Immunodominant epitopes of Hla **(A)**, IsdB **(B)**, MntC **(C)**, SEB **(D)** and LukG **(E)** were located on these structures using the PyMOL 1.1 program. The localizations of the human immunodominant epitopes Hla168–185, IsdB384–401, MntC55–72, SEB36–53 and LukG235–252on the 3D crystal structure of the antigens are shown in magenta.

Using available Protein Data Bank (PDB) crystal structures, we mapped the five epitopes onto Hla, SEB, MntC, IsdB, and LukG. Surface localization of all epitopes suggested high antibody accessibility (**Figure 4**). Hla_168–185_, IsdB_384–401_, SEB_37–54_, and LukG_235–252_ adopt β-sheet conformations, while MntC_55–72_ comprises a loop and α-helical elements.

**Figure 5:**
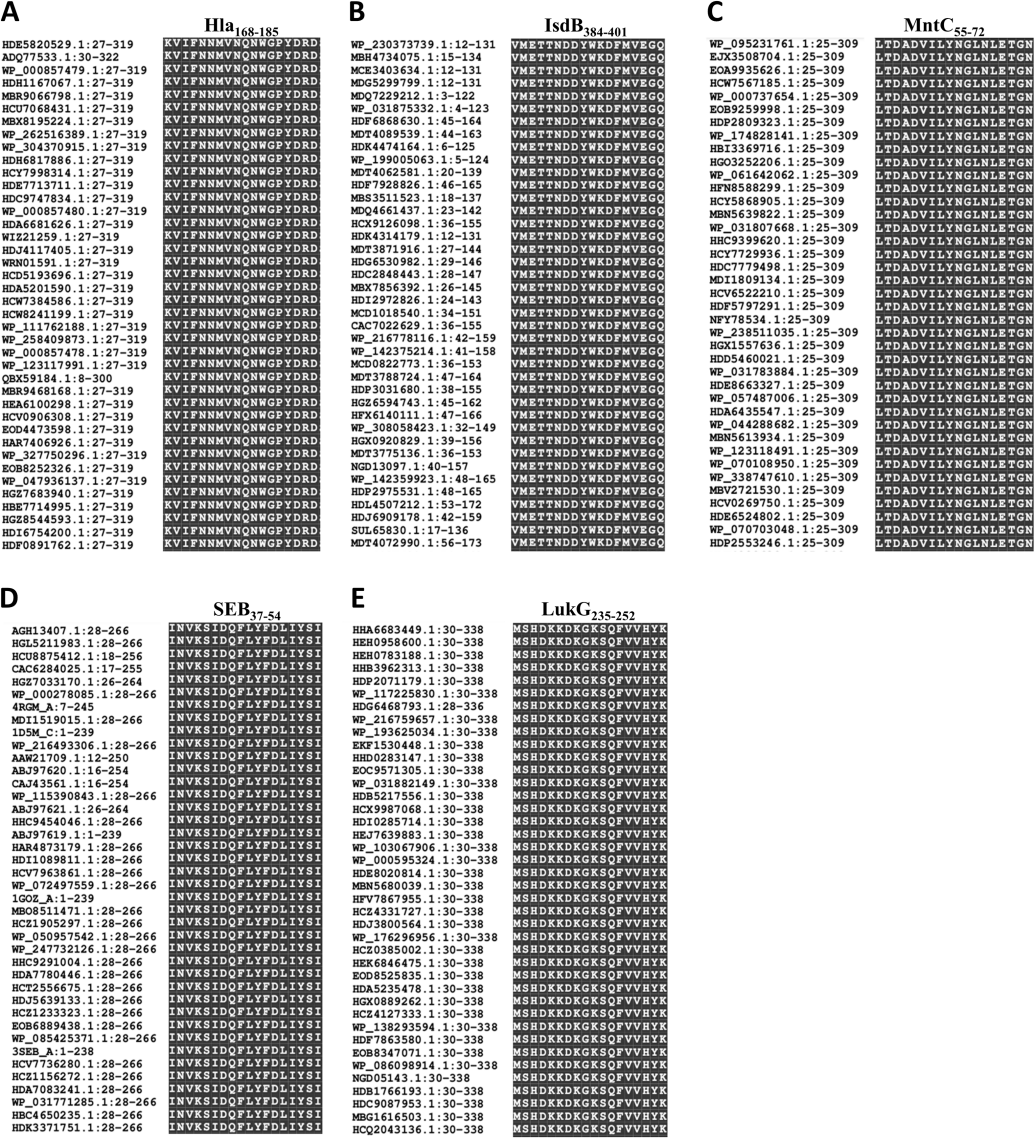
Sequence alignment of the immunodominant epitopes on Hla, SEB, MntC, IsdB and LukG. The sequences of Hla **(A)**, IsdB **(B)**, MntC **(C)**, SEB **(D)**, and LukG **(E)** from 40 different *S. aureus* strains were retrieved from the GenBank database. These sequences were aligned using the NCBI Basic Local Alignment Search Tool (BLAST) software.

The conservation of these immunodominant epitopes was determined by retrieving the amino acid sequences of Hla, SEB, MntC, IsdB, and LukG of 40 randomly selected *S. aureus* strains from the GenBank database for alignment. The sequences of all five immunodominant epitopes were completely conserved among the *S. aureu*s strains with 100% amino acid identity (**Figure 5**). Therefore, specific antibodies targeting these epitopes may cross-react with different *S. aureus* strains.

### 3.5 Combination of MIX3 and Quil-A improved infection survival against clinical MRSA isolates in lethal sepsis model

For the MIX3+Quil-A combination to have broad clinical utility against MRSA infections, it must exhibit protective efficacy against genetically divergent *S. aureus* lineages. Therefore, we selected previously reported three clinical isolates (BJ2, GZ9, CQ19) (33), which are phylogenetically representative and intersect with major internationally prevalent MRSA lineages. These three clinical isolates were collected from distinct regions across China and linked to diverse clinical manifestations, including pneumonia (BJ2), traumatic brain injury (GZ9), and septicaemia (CQ19). Notably, phylogenetic analysis of SEB gene sequences—conducted using MEGA 6.0 software via the neighbor-joining algorithm, with the maximum composite likelihood model for nucleotide distance calculation and bootstrap resampling for tree reliability validation—revealed that the selected strains (including MRSA252 and the three clinical isolates) not only represent phylogenetically distinct genetic backgrounds but also cluster with well-characterized international prevalent MRSA lineages such as N315, Mu50, and ST228.

**Figure 6:**
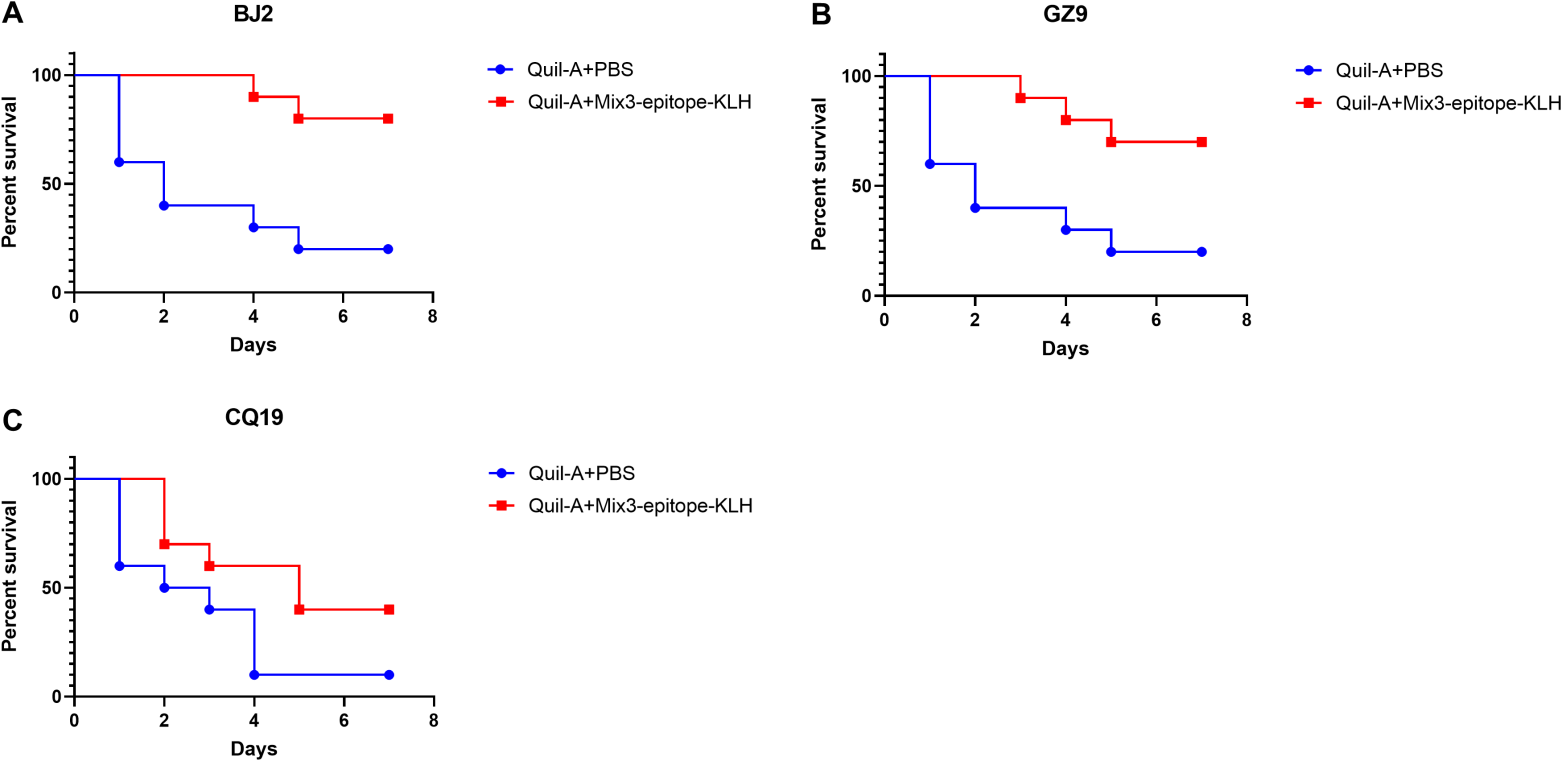
The multiple epitope vaccine induced protective response against clinical MRSA isolates infection in the immunized mice. **(A)** Percentage survival against BJ2 infection in the immunized mice and Quil-A + PBS-treated mice (n = 10). **(B)** Percentage survival against GZ9 infection in the immunized mice and Quil-A + PBS-treated mice (n = 10). **(C)** Percentage survival against CQ19 infection in the immunized mice and Quil-A + PBS-treated mice (n = 10).

Survival analysis following challenge with distinct clinical MRSA isolates revealed that MIX3-Quil-A vaccination significantly protected mice against all three clinical strains (**Figure 6**). Compared with the Quil-A + PBS group, the MIX3 + Quil-A group showed significant protection against the BJ2 (p = 0.0031), GZ9 (p = 0.0109), and CQ19 (p = 0.0572) clinical isolates.

### 3.6 Immunodominant epitope detection is effective in diagnosing clinical MRSA infection

We determined the diagnostic performance of the mixed immunodominant epitope peptides (i.e., the five core epitopes of Mix5, used in unconjugated form without KLH) assay by comparing its accuracy and reliability with that of the bacterial culture (gold standard) method. The results are summarized in **Table 2**. In total, 31 positive and 23 negative results were obtained from the bacterial cultures. The mixed immunodominant epitope peptide detection yielded 26 true positives, 4 false positives, 5 false negatives, and 19 true negatives. For inter-rater agreement analysis via the κ test, the inter-method consistency (between ELISA and culture) was quantified as κ = 0.661 (95% confidence interval [CI]: 0.459–0.863), with statistical significance (p < 0.001). Per the Landis-Koch criteria, a κ value ranging from 0.61 to 0.80 indicates “substantial agreement,” confirming good consistency between the two detection methods.

**Table 2:** Comparison of the results of mixed immunodominant epitope peptides (Hla_168–185_, IsdB_384–401_, MntC_55–72_, SEB_37–54_, and LukG_235–252_) detection and bacterial culture.

Mixed immunodominant epitopes detection	Bacterial culture (Gold standard)		Total	McNemar	Kappa	
	+	-			κ	*P*
+	26	4	30	*P* = 1	0.661	0.000
–	5	19	24			
Total	31	23				

For diagnostic purposes, KLH conjugate was omitted to avoid potential non-specific binding of anti-KLH antibodies in clinical sera, which could interfere with detection specificity.

The diagnostic performance of the mixed immunodominant epitope peptide detection was further evaluated using various indices (**Table 3**) as follows: sensitivity = 0.839 (indicating correct identification of 83.9% true positives) (95% CI, 0.684–0.926); specificity = 0.826 (indicating correct identification of 82.6% true negatives) (95% CI, 0.637–0.928); positive predictive value = 0.867 (95% CI, 0.712–0.945); negative predictive value = 0.792 (95% CI, 0.606–0.907); false negative rate = 0.161; false positive rate = 0.174; positive likelihood ratio = 4.823 (95% CI, 1.952–11.900), negative likelihood ratio = 0.195 (95% CI, 0.085–0.445); accuracy = 0.665; agreement rate = 0.833; and diagnostic odds ratio = 24.700 (indicating stronger diagnostic performance of mixed immunodominant epitope peptide detection than the bacterial culture). Given the inherent dependence of PPV and NPV on sample prevalence, we applied Bayes’ theorem to estimate these two indices under the scenario of a 52% clinical prevalence of MRSA. This prevalence was derived from a JAMA review, which reported that an estimated 52% of multidrug-resistant infections in hospitalized patients in the United States were caused by MRSA (11). The estimated values were as follows: PPV = 83.9% and NPV = 82.5%. These results suggest that mixed immunodominant epitope peptide detection is a reliable and accurate method for diagnosing bacterial infections with a high level of agreement with the gold standard bacterial culture method.

**Table 3:** Evaluation index data commonly used in diagnostic tests.

Evaluation index	Value
Sensitivity (Sen)	0.839
Specificity (Spe)	0.826
False Negative Rate (FNR)	0.161
False Positive Rate (FPR)	0.174
Positive Predictive Value (PPV)	0.867
Negative Predictive Value (NPV)	0.792
Positive Likelihood Ratio (PLR)	4.823
Negative Likelihood Ratio (NLR)	0.195
Accuracy (Acc)	0.665
Agreement Rate (AR)	0.833
Diagnostic Odds Ratio (DOR)	24.700

To further support these findings, we have supplemented the ROC curve (with AUC 0.842 and 95% CI 0.728–0.955) is presented in **Supplementary Figure S6A**, and calibration analysis is included therein; a scatter plot visualizing result consistency is shown in **Supplementary Figure S6B**. All 95% confidence intervals for sensitivity, specificity, PPV, and NPV are detailed in **Table 2**.

Thus, this diagnostic platform has dual clinical utility. It specifically detects clinical MRSA strains carrying target antigens. More importantly, it shows potential diagnostic value for toxin-mediated pathologies through its unique mechanism of capturing free toxin–antibody complexes. This dual diagnostic capacity substantially enhances the clinical applicability of this method for differentiating between invasive staphylococcal infections. Moreover, this method significantly shortens the clinical detection time window to 3 h (patient serum incubation with primary antibody, 1 h; secondary antibody incubation, 1 h; and color development and blocking steps, 1 h).

## 4 Discussion

Vaccination has successfully reduced infectious disease burden globally (39). However, antibiotic resistance-related deaths are the third leading cause of death worldwide (40); hence, the danger of multidrug-resistant bacteria such as MRSA should not be underestimated. Furthermore, no rapid diagnostic method that specifically targets MRSA are currently available for clinical use. Antibody response is essential for protection against infectious diseases (41). Therefore, the identification of targets that simultaneously confer preventive B cell-mediated immune protection and act as clinical diagnostic tools is of the utmost importance and would alleviate economic burden, especially in countries facing a severe MRSA threat.

Antigen-induced protective immunity primarily depends on eliciting specific immunodominant epitopes, thus making epitope localization crucial for elucidating antigenic protective mechanisms (42). We postulate that epitope studies should focus on the antigens of the four vaccine components (Hla, SEB, MntC, and IsdB) and LukG. Notably, Hla, SEB, MntC, IsdB, and LukG are MRSA autoantigens recognized by autoantibodies in the sera of infected patients, which indicates that the epitopes identified using patient sera may exhibit enhanced specificity. In the present study, we found a significant difference in the response to *S. aureus* infection between mice immunized with Hla, SEB, MntC, IsdB, and LukG immunodominant epitopes and control mice in the bacteremia model, showing the favorable protective properties of Hla, SEB, MntC, IsdB, and LukG immunodominant epitopes. As expected, the epitope-specific antibody titers correlated with survival, which is consistent with previous findings that humoral immune responses are essential for Hla, SEB, and LukG-mediated protection (43). Additionally, Quil-A adjuvant supplementation compensated for the deficiency of B-cell immunodominant epitopes in stimulating cellular immunity.

SEB is a MRSA-secreted superantigen; hence, a small quantity activates a large number of T cells, which secrete inflammatory cytokines such as IFN-γ and TNF-α that induce tissue and organ damage while simultaneously disrupting the normal activity of the immune system by inducing immune tolerance (44). The whole SEB antigen and its dominant epitopes counteract SEB-producing MRSA infections (33), making it a good vaccine candidate. Hla is a pathogenic exotoxin with hemolytic activity secreted by *S. aureus*. It binds to the ADAM10 receptor and specifically disrupts the physiological barrier function of the vascular endothelium (45). Additionally, it plays vital roles in bacteremia caused by *S. aureus* infection and migratory foci of infection. Hla antibody levels correlated positively with survival in patients with *S. aureus* (46). Leukocidins target and kill large numbers of human primary leukocytes that are essential for innate immune defense and adaptive immunity (47). They are a key virulence factor used by *S. aureus* to counteract immune defenses. Hence, host-mediated protection may be augmented by targeting leukocidin cytokine-mediated immune evasion through inoculation (19). MntC occurs abundantly on the cell membrane surface and contributes significantly to defense against oxidative stress in organisms (48). Additionally, antibodies targeting MntC and MntC-specific Th17 cells collaborate effectively in preventing *S. aureus–*induced infections (16). The IsdB protein is consistently present in *S. aureus*. It triggers a strong immune response that is associated with increased resistance in mouse infection models (49). Furthermore, Th17 cells that produce IL-17A are crucial for the protective effects of IsdB-based vaccines against severe *S. aureus* infections in mice (17). Therefore, identifying and using the immunodominant epitopes of these five important *S. aureus* autoantigens is a reasonable approach for integrated diagnosis and treatment.

The previously identified Hla, SEB, MntC, IsdB, and LukG immunodominant epitopes are Hla_42–59_, Hla_84–101_, Hla_186–203_, Hla_109–126_, Hla_157–174_, and Hla_193–210_ (34, 35); SEB_31–48_, SEB_83–92_, SEB_97–114_, SEB_133–150_, SEB_193–210_, SEB_205–222_, and SEB_247–261_ (15, 33, 50, 51); LukG_64–75_, LukG_199–216_, and LukG_262–269_ (52); MntC_7–24_, MntC_1–60_, MntC_120–160_, and MntC_220–260_ (18, 53); and IsdB_402–419_ and IsdB_432–449_ (34). Although conventional investigations have predominantly relied on animal immunization models and mixed-antigen-immunized cohorts, they lack MRSA-specific convalescent-phase models. We bridged this gap by systematically using an overlapping peptide ELISA to characterize linear B-cell epitopes of the five antigens in the convalescent sera of patients with clinical MRSA-induced sepsis and identifying 10 novel linear immunodominant peptides. Homology analysis of the sequences showed that these immunodominant epitopes are highly conserved across *S. aureus* strains, suggesting that these epitopes are qualified candidates for vaccine development and that they may provide cross-immune responses against a wide range of *S. aureus* isolates. This finding was corroborated by results of survival analysis performed using genetically diverse clinical isolates. Additionally, T-cell epitope prediction has shown that some of the identified epitopes may act as potential CTL- and Th-cell epitopes (**Supplementary Table S1**). Moreover, the protective efficacy of toxin-neutralizing antibodies against *S. aureus* infections has been established previously (54). This finding potentially explains the reason for the superior protective efficacy of the three-toxin epitope component vaccine compared to that of the five-epitope component vaccine, with potential contributing factors including competition for T cell help, epitope density on KLH, physical interference, and antigen dose effects. Notably, DNASTAR-based B cell epitope prediction showed a partial overlap with the experimentally validated epitopes (**Supplementary Table S1** and **Supplementary Figures S1**-**S5**), highlighting the complementary yet imperfect nature of *in silico* prediction tools because conformational epitopes, post-translational modifications, and host-specific glycosylation patterns are often overlooked (55). Although bioinformatics provides valuable guidance for epitope prioritization, these findings highlight the critical need for orthogonal experimental validation to mitigate the false positives/negatives inherent in sequence-based predictions.

Bacterial culture is widely recognized as the gold standard for diagnosing bacterial infections (56) because the enrichment of bacteria in blood culture bottles containing enriched liquid culture media enables the growth and multiplication of pathogens even in the presence of residual antibiotics. However, limitations of the method include its time-consuming nature, the inability to culture all microbes, and challenges associated with slow-growing or fastidious bacteria (57). In contrast, the immunodominant epitope peptide diagnostic method has advantages such as faster diagnosis and ability to be prepared into polypeptide chips to develop high-throughput, miniaturized, and automated bioassay technology for detecting *S. aureus* infection. Its diagnostic performance and clinical relevance are further highlighted by key metrics: a PLR of 4.823, which increases the post-test probability of MRSA infection by approximately 4.8-fold and strongly reinforces diagnostic suspicion of infection; and a NLR of 0.195, which reduces the post-test probability of MRSA infection by over 80% and reliably rules out infection. Notably, likelihood ratios are less influenced by disease prevalence than other metrics, enhancing the assay’s generalizability across different clinical settings. However, this approach is not effective in detecting resistance genes; nevertheless, the speed of bacterial identification is commendable. This balance of strengths and limitations solidifies the assay’s role as a complementary diagnostic tool—one with particular value in resource-limited settings where access to molecular testing platforms is restricted.

Notably, by performing consistency alignment between the epitope sequences screened in this study and the homologous sequences of currently internationally prevalent *S. aureus* strains (e.g., ST59, ST398, USA300, N315, Mu50, etc.), the results showed a high sequence consistency rate between them (**Table 4**). This finding can partially explain why the detection reagents constructed based on these epitopes exhibit high diagnostic efficacy, and the immune compositions centered on these epitopes can provide good immune protection against strains from different sources. The above results further confirm that the epitopes screened in this study have excellent diagnostic application potential and immune protection value, providing key experimental evidence for the subsequent development of accurate diagnostic tools for *S. aureus* infections and the design of multi-epitope vaccines.

**Table 4:** Consistency alignment results between screened immunodominant epitopes and relevant virulence protein sequences of internationally prevalent *S. aureus* strains.

Strain	Type	The immunodominant epitopes	Sequence of the immunodominant epitopes	Identity rate
USA300	ST8	Hla_168-185_	KVIFNNMVNQNWGPYDRD	100%
		IsdB_384-401_	VMETTNDDYWKDFMVEGQ	100%
		MntC_55-72_	LTDADVILYNGLNLETGN	100%
		SEB_37-54_	/	/
		LukG_235-252_	MSHDKKDKGKSQFVVHYK	100%
N315	ST5	Hla_168-185_	KVIFNNMVNQNWGPYDRD	100%
		IsdB_384-401_	VMETTNDDYWKDFMVEGQ	100%
		MntC_55-72_	LTDADVILYNGLNLETGN	100%
		SEB_37-54_	INVKSIDQFLYFDLIYSI	100%
		LukG_235-252_	MSHDKKDKGKSQFVVHYK	100%
Mu50	ST239	Hla_168-185_	KVIFNNMVNQNWGPYDRD	100%
		IsdB_384-401_	VMETTNDDYWKDFMVEGQ	100%
		MntC_55-72_	LTDADVILYNGLNLETGN	100%
		SEB_37-54_	INVKSIDQFLYFDLIYSI	100%
		LukG_235-252_	MSHDKKDKGKSQFVVHYK	100%
M013	ST59	Hla_168-185_	KVIFNNMVNQNWGPYDRD	100%
		IsdB_384-401_	VMETTNDDYWKDFMVEGQ	100%
		MntC_55-72_	LTDADVILYNGLNLETGN	100%
		SEB_37-54_	INVKSIDQFLYFDLIYSI	100%
		LukG_235-252_	MSHDKKDEGKSKFVVHYK	88.9%
LA-MRSA ST398	ST398	Hla_168-185_	KVIFNNMVNQNWGPYDRD	100%
		IsdB_384-401_	VMETTNDDYWKDFMVEGQ	100%
		MntC_55-72_	LTDADVILYNGLNLETGN	100%
		SEB_37-54_	/	/
		LukG_235-252_	MSHDKKDEGKSKFVVHYK	88.9%

The USA300 strain and livestock-associated methicillin-resistant *Staphylococcus aureus* (LA-MRSA) ST398 do not express SEB protein.

In conclusion, we have identified 10 B-cell immunodominant epitopes from five antigens (Hla, SEB, MntC, IsdB, and LukG) using the convalescent serum of patients infected with MRSA. Among these, five elicited partial protective immune responses. The combination of these epitopes showed broad-spectrum protection and potential for use as diagnostic biomarkers. Furthermore, bioinformatic analysis indicated that these epitopes are potential dual T–B cell targets for future MRSA vaccine development. In future studies, we plan to perform a functional characterization of the T-cell responses to these epitopes, simultaneously develop epitope-specific monoclonal antibodies, validate their protective efficacy in different infection models, and optimize their diagnostic performance by developing a peptide microarray platform to enhance assay sensitivity and specificity.

## Data Availability

The datasets presented in this study can be found in online repositories. The names of the repository/repositories and accession number(s) can be found in the article/**Supplementary Material**.

## References

[1] GBD 2019 Antimicrobial Resistance Collaborators. Global mortality associated with 33 bacterial pathogens in 2019: a systematic analysis for the Global Burden of Disease Study 2019. Lancet (London England). (2022) 400:2221–48. doi: 10.1016/S0140-6736(22)02185-7, PMID: 36423648 PMC9763654

[2] Cheung GYC Bae JS Otto M. Pathogenicity and virulence of Staphylococcus aureus. Virulence. (2021) 12:547–69. doi: 10.1080/21505594.2021.1878688, PMID: 33522395 PMC7872022

[3] Cassini A Högberg LD Plachouras D Quattrocchi A Hoxha A Simonsen GS. Attributable deaths and disability-adjusted life-years caused by infections with antibiotic-resistant bacteria in the EU and the European Economic Area in 2015: a population-level modelling analysis. Lancet Infect Dis. (2019) 19:56–66. doi: 10.1016/S1473-3099(18)30605-4, PMID: 30409683 PMC6300481

[4] Kavanagh KT. Control of MSSA and MRSA in the United States: protocols, policies, risk adjustment and excuses. Antimicrob Resist Infect Control. (2019) 8:103. doi: 10.1186/s13756-019-0550-2, PMID: 31244994 PMC6582558

[5] Wang B Xu Y Zhao H Wang X Rao L Guo Y. Methicillin-resistant Staphylococcus aureus in China: a multicentre longitudinal study and whole-genome sequencing. Emerg Microbes Infect. (2022) 11:532–42. doi: 10.1080/22221751.2022.2032373, PMID: 35060838 PMC8843102

[6] Brown NM Brown EM. Treatment of methicillin-resistant Staphylococcus aureus (MRSA): updated guidelines from the UK. J Antimicrob Chemother. (2021) 76:1377–8. doi: 10.1093/jac/dkab036, PMID: 33582806

[7] Tsuzuki S Matsunaga N Yahara K Shibayama K Sugai M Ohmagari N. Disease burden of bloodstream infections caused by antimicrobial-resistant bacteria: A population-level study, Japn 2015-2018. Int J Infect Dis. (2021) 108:119–24. doi: 10.1016/j.ijid.2021.05.018, PMID: 33992765

[8] Minter DJ Appa A Chambers HF Doernberg SB. Contemporary management of staphylococcus aureus bacteremia-controversies in clinical practice. Clin Infect Dis. (2023) 77:e57–68. doi: 10.1093/cid/ciad500, PMID: 37950887 PMC11959183

[9] Chand U Priyambada P Kushawaha PK. Staphylococcus aureus vaccine strategy: Promise and challenges. Microbiol Res. (2023) 271:127362. doi: 10.1016/j.micres.2023.127362, PMID: 36958134

[10] Adamu Y Puig-Asensio M Dabo B Schweizer ML. Comparative effectiveness of daptomycin versus vancomycin among patients with methicillin-resistant Staphylococcus aureus (MRSA) bloodstream infections: A systematic literature review and meta-analysis. PLoS One. (2024) 19:e0293423. doi: 10.1371/journal.pone.0293423, PMID: 38381737 PMC10881006

[11] Tong SYC Fowler VG Jr. Skalla L Holland TL. Management of staphylococcus aureus bacteremia: A review. Jama. (2025) 334:798–808. doi: 10.1001/jama.2025.4288, PMID: 40193249 PMC12663922

[12] Lowy FD. Staphylococcus aureus infections. N Engl J Med. (1998) 339:520–32. doi: 10.1056/NEJM199808203390806, PMID: 9709046

[13] Jiang XY Gong MQ Zhang HJ Peng AQ Xie Z Sun D. The safety and immunogenicity of a recombinant five-antigen Staphylococcus aureus vaccine among patients undergoing elective surgery for closed fractures: A randomized, double-blind, placebo-controlled, multicenter phase 2 clinical trial. Vaccine. (2023) 41:5562–71. doi: 10.1016/j.vaccine.2023.07.047, PMID: 37516573

[14] Bubeck Wardenburg J Schneewind O. Vaccine protection against Staphylococcus aureus pneumonia. J Exp Med. (2008) 205:287–94. doi: 10.1084/jem.20072208, PMID: 18268041 PMC2271014

[15] Zhao Z Li B Sun HQ Zhang JY Wang YL Chen L. Fine-mapping of immunodominant linear B-cell epitopes of the Staphylococcus aureus SEB antigen using short overlapping peptides. PLoS One. (2014) 9:e90445. doi: 10.1371/journal.pone.0090445, PMID: 24599257 PMC3943954

[16] Yu W Yao D Yu S Wang X Li X Wang M. Protective humoral and CD4(+) T cellular immune responses of Staphylococcus aureus vaccine MntC in a murine peritonitis model. Sci Rep. (2018) 8:3580. doi: 10.1038/s41598-018-22044-y, PMID: 29483570 PMC5832154

[17] Joshi A Pancari G Cope L Bowman EP Cua D Proctor RA. Immunization with Staphylococcus aureus iron regulated surface determinant B (IsdB) confers protection *via* Th17/IL17 pathway in a murine sepsis model. Hum Vaccines immunotherapeutics. (2012) 8:336–46. doi: 10.4161/hv.18946, PMID: 22327491 PMC3426080

[18] Zeng H Zhang J Song X Zeng J Yuan Y Chen Z. An immunodominant epitope-specific monoclonal antibody cocktail improves survival in a mouse model of staphylococcus aureus bacteremia. J Infect Dis. (2021) 223:1743–52. doi: 10.1093/infdis/jiaa602, PMID: 32959055

[19] Tam K Lacey KA Devlin JC Coffre M Sommerfield A Chan R. Targeting leukocidin-mediated immune evasion protects mice from Staphylococcus aureus bacteremia. J Exp Med. (2020) 217:e20190541. doi: 10.1084/jem.20190541, PMID: 32602902 PMC7478724

[20] Alonzo F Torres VJ 3rd. The bicomponent pore-forming leucocidins of Staphylococcus aureus. Microbiol Mol Biol Rev. (2014) 78:199–230. doi: 10.1128/MMBR.00055-13, PMID: 24847020 PMC4054254

[21] DuMont AL Yoong P Surewaard BG Benson MA Nijland R van Strijp JA. Staphylococcus aureus elaborates leukocidin AB to mediate escape from within human neutrophils. Infect Immun. (2013) 81:1830–41. doi: 10.1128/IAI.00095-13, PMID: 23509138 PMC3648020

[22] Yanai M Rocha MA Matolek AZ Chintalacharuvu A Taira Y Chintalacharuvu K. Separately or combined, LukG/LukH is functionally unique compared to other staphylococcal bicomponent leukotoxins. PLoS One. (2014) 9:e89308. doi: 10.1371/journal.pone.0089308, PMID: 24586678 PMC3930693

[23] Chen X Missiakas D. Novel antibody-based protection/therapeutics in staphylococcus aureus. Annu Rev Microbiol. (2024) 78:425–46. doi: 10.1146/annurev-micro-041222-024605, PMID: 39146354

[24] Luo L Li Q Xing C Li C Pan Y Sun H. Antibody-based therapy: An alternative for antimicrobial treatment in the post-antibiotic era. Microbiological Res. (2025) 290:127974. doi: 10.1016/j.micres.2024.127974, PMID: 39577369

[25] Correia BE Bates JT Loomis RJ Baneyx G Carrico C Jardine JG. Proof of principle for epitope-focused vaccine design. Nature. (2014) 507:201–6. doi: 10.1038/nature12966, PMID: 24499818 PMC4260937

[26] Akram A Inman RD. Immunodominance: a pivotal principle in host response to viral infections. Clin Immunol. (2012) 143:99–115. doi: 10.1016/j.clim.2012.01.015, PMID: 22391152

[27] Kolla HB Makam SS Reddy PN. Mapping of conserved immunodominant epitope peptides in the outer memb rane porin (Omp) L of prominent Enterobacteriaceae pathogens associate d with gastrointestinal infections. J Genet Eng Biotechnol. (2023) 21:146. doi: 10.1186/s43141-023-00622-6, PMID: 38012455 PMC10682294

[28] Mayer RL Verbeke R Asselman C Aernout I Gul A Eggermont D. Immunopeptidomics-based design of mRNA vaccine formulations against Listeria monocytogenes. Nat Commun. (2022) 13:6075. doi: 10.1038/s41467-022-33721-y, PMID: 36241641 PMC9562072

[29] He T Zhang F Zhang J Wei S Ning J Yuan H. UreB immunodominant epitope-specific CD8(+) T-cell responses were beneficial in reducing gastric symptoms in Helicobacter pylori-infected individuals. Helicobacter. (2023) 28:e12959. doi: 10.1111/hel.12959, PMID: 36828665

[30] Aw-Yong KL Sam IC Koh MT Chan YF. Immunodominant igM and igG epitopes recognized by antibodies induced in enterovirus A71-associated hand, foot and mouth disease patients. PLoS One. (2016) 11:e0165659. doi: 10.1371/journal.pone.0165659, PMID: 27806091 PMC5091889

[31] Liu Q Zhao H Li Z Zhang Z Huang R Gu M. Broadly neutralizing antibodies derived from the earliest COVID-19 convalescents protect mice from SARS-CoV-2 variants challenge. Signal transduction targeted Ther. (2023) 8:347. doi: 10.1038/s41392-023-01615-0, PMID: 37704615 PMC10499932

[32] Rahman KS Darville T Russell AN O'Connell CM Wiesenfeld HC Hillier SL. Discovery of human-specific immunodominant chlamydia trachomatis B cell epitopes. mSphere. (2018) 3:e00246-18. doi: 10.1128/mSphere.00246-18, PMID: 30068558 PMC6070735

[33] Zhao Z Sun HQ Wei SS Li B Feng Q Zhu J. Multiple B-cell epitope vaccine induces a Staphylococcus enterotoxin B-specific IgG1 protective response against MRSA infection. Sci Rep. (2015) 5:12371. doi: 10.1038/srep12371, PMID: 26201558 PMC4511869

[34] Chen Z Gou Q Xiong Q Duan L Yuan Y Zhu J. Immunodominance of epitopes and protective efficacy of HI antigen are differentially altered using different adjuvants in a mouse model of staphylococcus aureus bacteremia. Front Immunol. (2021) 12:684823. doi: 10.3389/fimmu.2021.684823, PMID: 34122448 PMC8190387

[35] Wei J Cheng X Zhang Y Gao C Wang Y Peng Q. Identification and application of a neutralizing epitope within alpha-hemolysin using human serum antibodies elicited by vaccination. Mol Immunol. (2021) 135:45–52. doi: 10.1016/j.molimm.2021.03.028, PMID: 33873093

[36] Adelusi TI Ogunlana AT Oyewole MP Ojo TO Olaoba OT Oladipo EK. Designing of an innovative conserved multiepitope subunit vaccine targeting SARS-CoV-2 glycoprotein and nucleoprotein through immunoinformatic. Sci Rep. (2025) 15:2563. doi: 10.1038/s41598-024-72495-9, PMID: 39833186 PMC11747174

[37] Ferraro A Buonocore SM Auquier P Nicolas I Wallemacq H Boutriau D. Role and plasticity of Th1 and Th17 responses in immunity to Staphylococcus aureus. Hum Vaccines immunotherapeutics. (2019) 15:2980–92. doi: 10.1080/21645515.2019.1613126, PMID: 31149870 PMC6930085

[38] Gupta SK Parlane N Bridgeman B Lynch AT Dangerfield EM Timmer MSM. The trehalose glycolipid C18Brar promotes antibody and T-cell immune responses to Mannheimia haemolytica and Mycoplasma ovipneumoniae whole cell antigens in sheep. PLoS One. (2023) 18:e0278853. doi: 10.1371/journal.pone.0278853, PMID: 36656850 PMC9851559

[39] Vaccines work. Nat Commun. (2018) 9:1666. doi: 10.1038/s41467-018-04085-z, PMID: 29691393 PMC5915378

[40] Antimicrobial Resistance Collaborators. Global burden of bacterial antimicrobial resistance in 2019: a systematic analysis. Lancet. (2022) 399:629–55. doi: 10.1016/S0140-6736(21)02724-0, PMID: 35065702 PMC8841637

[41] Otsubo R Yasui T. Monoclonal antibody therapeutics for infectious diseases: Beyond normal human immunoglobulin. Pharmacol Ther. (2022) 240:108233. doi: 10.1016/j.pharmthera.2022.108233, PMID: 35738431 PMC9212443

[42] Pollard AJ Bijker EM. A guide to vaccinology: from basic principles to new developments. Nat Rev Immunol. (2021) 21:83–100. doi: 10.1038/s41577-020-00479-7, PMID: 33353987 PMC7754704

[43] Zhang J Yang F Zhang X Jing H Ren C Cai C. Protective efficacy and mechanism of passive immunization with polyclonal antibodies in a sepsis model of staphylococcus aureus infection. Sci Rep. (2015) 5:15553. doi: 10.1038/srep15553, PMID: 26490505 PMC4614693

[44] Fries BC Varshney AK. Bacterial toxins-staphylococcal enterotoxin B. Microbiol Spectr. (2013) 1:10.1128/microbiolspec.AID-0002-2012. doi: 10.1128/microbiolspec.AID-0002-2012, PMID: 26184960 PMC5086421

[45] Kwiecinski JM Horswill AR. Staphylococcus aureus bloodstream infections: pathogenesis and regulatory mechanisms. Curr Opin Microbiol. (2020) 53:51–60. doi: 10.1016/j.mib.2020.02.005, PMID: 32172183 PMC7244392

[46] Sharma-Kuinkel BK Wu Y Tabor DE Mok H Sellman BR Jenkins A. Characterization of alpha-toxin hla gene variants, alpha-toxin expression levels, and levels of antibody to alpha-toxin in hemodialysis and postsurgical patients with Staphylococcus aureus bacteremia. J Clin Microbiol. (2015) 53:227–36. doi: 10.1128/JCM.02023-14, PMID: 25392350 PMC4290928

[47] Spaan AN van Strijp JAG Torres VJ. Leukocidins: staphylococcal bi-component pore-forming toxins find their receptors. Nat Rev Microbiol. (2017) 15:435–47. doi: 10.1038/nrmicro.2017.27, PMID: 28420883 PMC5621924

[48] Coady A Xu M Phung Q Cheung TK Bakalarski C Alexander MK. The staphylococcus aureus ABC-type manganese transporter mntABC is critical for reinitiation of bacterial replication following exposure to phagocytic oxidative burst. PLoS One. (2015) 10:e0138350. doi: 10.1371/journal.pone.0138350, PMID: 26379037 PMC4574778

[49] Kuklin NA Clark DJ Secore S Cook J Cope LD McNeely T. A novel Staphylococcus aureus vaccine: iron surface determinant B induces rapid antibody responses in rhesus macaques and specific increased survival in a murine S. aureus sepsis model. Infection Immun. (2006) 74:2215–23. doi: 10.1128/IAI.74.4.2215-2223.2006, PMID: 16552052 PMC1418914

[50] Kum WW Chow AW. Inhibition of staphylococcal enterotoxin A-induced superantigenic and lethal activities by a monoclonal antibody to toxic shock syndrome toxin-1. J Infect Dis. (2001) 183:1739–48. doi: 10.1086/320732, PMID: 11372026

[51] Turner KB Zabetakis D Legler P Goldman ER Anderson GP. Isolation and epitope mapping of staphylococcal enterotoxin B single-domain antibodies. Sensors (Basel). (2014) 14:10846–63. doi: 10.3390/s140610846, PMID: 24949641 PMC4118376

[52] Badarau A Rouha H Malafa S Battles MB Walker L Nielson N. Context matters: The importance of dimerization-induced conformation of the LukGH leukocidin of Staphylococcus aureus for the generation of neutralizing antibodies. mAbs. (2016) 8:1347–60. doi: 10.1080/19420862.2016.1215791, PMID: 27467113 PMC5058624

[53] Ahmadi K Pouladfar G Kalani M Faezi S Pourmand MR Hasanzadeh S. Epitope-based immunoinformatics study of a novel Hla-MntC-SACOL0723 fusion protein from Staphylococcus aureus: Induction of multi-pattern immune responses. Mol Immunol. (2019) 114:88–99. doi: 10.1016/j.molimm.2019.05.016, PMID: 31351414

[54] Karauzum H Venkatasubramaniam A Adhikari RP Kort T Holtsberg FW Mukherjee I. IBT-V02: A multicomponent toxoid vaccine protects against primary and secondary skin infections caused by staphylococcus aureus. Front Immunol. (2021) 12:624310. doi: 10.3389/fimmu.2021.624310, PMID: 33777005 PMC7987673

[55] Galanis KA Nastou KC Papandreou NC Petichakis GN Pigis DG Iconomidou VA. Linear B-cell epitope prediction for in silico vaccine design: A performance review of methods available *via* command-line interface. Int J Mol Sci. (2021) 22:624310. doi: 10.3390/ijms22063210, PMID: 33809918 PMC8004178

[56] Templier V Livache T Boisset S Maurin M Slimani S Mathey R. Biochips for direct detection and identification of bacteria in blood culture-like conditions. Sci Rep. (2017) 7:9457. doi: 10.1038/s41598-017-10072-z, PMID: 28842712 PMC5572712

[57] Lewis WH Tahon G Geesink P Sousa DZ Ettema TJG. Innovations to culturing the uncultured microbial majority. Nat Rev Microbiol. (2021) 19:225–40. doi: 10.1038/s41579-020-00458-8, PMID: 33093661

