# Supplementary material for: Integrated immunodominant epitope discovery for dual-purpose rapid and economical diagnostic and immunoprotective applications against MRSA
Source: Front Immunol. 2025 Oct 20;16:1697829. doi: 10.3389/fimmu.2025.1697829 (PMC12580254; doi:10.3389/fimmu.2025.1697829)
Supplement: Supplementary file 7 [file Table1.docx]

Table S1 Correspondence between epitopes identified in this study and T/B-cell epitope prediction results

| Immunodominant epitope | Predicted CTL epitope | |  | Predicted Th cell epitope | |  | Predicted B cell epitope |
| --- | --- | --- | --- | --- | --- | --- | --- |
|  | Human | Mouse |  | Human | Mouse |  |  |
| Hla_168-185_ | - | - |  | + | - |  | + |
| SEB_37-54_ | + | + |  | + | + |  | - |
| LukG_235-252_ | + | - |  | + | + |  | + |
| IsdB_384-401_ | - | - |  | + | - |  | + |
| MntC_55-72_ | + | - |  | + | + |  | - |
