# Supplementary material for: Integrated immunodominant epitope discovery for dual-purpose rapid and economical diagnostic and immunoprotective applications against MRSA
Source: Front Immunol. 2025 Oct 20;16:1697829. doi: 10.3389/fimmu.2025.1697829 (PMC12580254; doi:10.3389/fimmu.2025.1697829)
Supplement: Supplementary file 8 [file Table2.docx]

Table S2 Predicted immunodominant cytotoxic T lymphocyte (CTL) epitopes of Hla protein

| Phenotypic classification | Position of the initial amino acid | Sequence | SYFPEITHI（Score） | NetCTL（COMB） |
| --- | --- | --- | --- | --- |
| HLA-A2 * 0201 | 134 | GLIGANVSI | 27 | 1.1764 |
|  | 78 | LAWPSAFKV | 21 | 1.0203 |
|  | 59 | GTIAGQYRV | 20 | 0.8611 |
| HLA-A3 * 0301 | 77 | GLAWPSAFK | 24 | 1.7371 |
|  | 139 | NVSIGHTLK | 24 | 0.9806 |
|  | 155 | TILESPTDK | 23 | 1.0141 |
|  | 156 | ILESPTDKK | 22 | 1.0518 |
|  | 232 | ITMDRKATK | 22 | 1.2065 |
|  | 60 | TIAGQYRVY | 21 | 0.8469 |
|  | 110 | KEYMSTLTY | 20 | 1.0296 |
| HLA-B7 * 0702 | 80 | WPSAFKVQL | 25 | 1.3964 |
|  | 212 | DPNKASSLL | 21 | 0.9086 |
|  | 225 | SPDFATVIT | 20 | 0.8690 |
| H2-Db | 240 | KQQTNIDVI | 25 |  |
|  | 70 | EEGANKSGL | 21 |  |
|  | 224 | FSPDFATVI | 21 |  |
| H2-Kd | 27 | TYDKENGML | 22 |  |
| H2-Kk | 30 | KENGMLKKV | 24 |  |
|  | 44 | DDKNHNKKI | 23 |  |
|  | 157 | LESPTDKKV | 23 |  |
|  | 161 | TDKKVGWKV | 21 |  |
|  | 12 | TDIGSNTTV | 20 |  |
|  | 99 | SDYYPRNSI | 20 |  |
| H2-Ld | 80 | WPSAFKVQL | 23 |  |
|  | 188 | NPVYGNQLF | 22 |  |
|  | 202 | GSMKAAENF | 22 |  |
|  | 212 | DPNKASSLL | 21 |  |
|  | 216 | ASSLLSSGF | 20 |  |
|  | 220 | LSSGFSPDF | 20 |  |
