# Supplementary material for: Integrated immunodominant epitope discovery for dual-purpose rapid and economical diagnostic and immunoprotective applications against MRSA
Source: Front Immunol. 2025 Oct 20;16:1697829. doi: 10.3389/fimmu.2025.1697829 (PMC12580254; doi:10.3389/fimmu.2025.1697829)
Supplement: Supplementary file 9 [file Table3.docx]

Table S3 Predicted immunodominant Helper T cell epitopes of Hla protein

| Phenotypic classification | Position of the initial amino acid | Sequence | SYFPEITHI（Score） | NetMHC Ⅱ pan 4.0（Rank） |
| --- | --- | --- | --- | --- |
| HLA-DRB1*0101 | 184 | RDSWNPVYGNQLFMK | 33 | 0.76 |
|  | 129 | SGKIGGLIGANVSIG | 32 | 0.93 |
|  | 65 | YRVYSEEGANKSGLA | 28 | 1.70 |
|  | 164 | KVGWKVIFNNMVNQN | 25 | 0.61 |
|  | 130 | GKIGGLIGANVSIGH | 24 | 2.62 |
| HLA-DRB1*0301 | 146 | LKYVQPDFKTILESP | 35 | 0.33 |
|  | 23 | GDLVTYDKENGMLKK | 29 | 0.42 |
|  | 229 | ATVITMDRKATKQQT | 29 | 0.02 |
|  | 249 | YERVRDDYQLHWTST | 28 | 4.09 |
|  | 39 | FYSFIDDKNHNKKIL | 27 | 3.27 |
| HLA-DRB1*0401 | 164 | KVGWKVIFNNMVNQN | 28 | 0.93 |
|  | 36 | KKVFYSFIDDKNHNK | 22 | 1.25 |
|  | 65 | YRVYSEEGANKSGLA | 22 | 0.90 |
|  | 150 | QPDFKTILESPTDKK | 22 | 4.14 |
|  | 246 | DVIYERVRDDYQLHW | 22 | 2.54 |
|  | 64 | QYRVYSEEGANKSGL | 20 | 1.26 |
|  | 113 | MSTLTYGFNGNVTGD | 20 | 4.74 |
|  | 201 | NGSMKAAENFLDPNK | 20 | 2.29 |
|  | 165 | VGWKVIFNNMVNQNW | 18 | 4.77 |
| HLA-DRB1*0701 | 109 | TKEYMSTLTYGFNGN | 32 | 0.63 |
|  | 193 | NQLFMKTRNGSMKAA | 32 | 1.63 |
|  | 164 | KVGWKVIFNNMVNQN | 30 | 1.43 |
|  | 49 | NKKILVIRTKGTIAG | 22 | 3.04 |
|  | 51 | KILVIRTKGTIAGQY | 20 | 1.92 |
|  | 184 | RDSWNPVYGNQLFMK | 18 | 0.52 |
| HLA-DRB1*1101 | 48 | HNKKILVIRTKGTIA | 21 | 4.53 |
|  | 192 | GNQLFMKTRNGSMKA | 21 | 1.72 |
|  | 22 | TGDLVTYDKENGMLK | 20 | 4.29 |
|  | 58 | KGTIAGQYRVYSEEG | 20 | 0.97 |
|  | 228 | FATVITMDRKATKQQ | 20 | 0.99 |
| HLA-DRB1*1501 | 95 | VAQISDYYPRNSIDT | 28 | 0.947 |
|  | 162 | DKKVGWKVIFNNMVN | 20 | 4.57 |
|  | 49 | NKKILVIRTKGTIAG | 18 | 2.45 |
| H2-Ad | 87 | YDAIVKVHVKTIDYD | 25 |  |
|  | 10 | YVVYESVENNESMMD | 24 |  |
|  | 5 | LQDTKYVVYESVENN | 20 |  |
| H2-Ed | 101 | DGQYHVRIVDKEAFT | 26 | 2.88 |
|  | 105 | HVRIVDKEAFTKANL | 20 | 2 |
| H2-Au | 10 | YVVYESVENNESMMD |  | 4.42 |
| H2-Ek | 59 | GQRVRTISKDAKNNT | 24 | 0.30 |
