# Supplementary material for: Integrated immunodominant epitope discovery for dual-purpose rapid and economical diagnostic and immunoprotective applications against MRSA
Source: Front Immunol. 2025 Oct 20;16:1697829. doi: 10.3389/fimmu.2025.1697829 (PMC12580254; doi:10.3389/fimmu.2025.1697829)
Supplement: Supplementary file 10 [file Table4.docx]

Table S4 Predicted immunodominant B cell epitopes of Hla protein

| No. | Amino acid position | Sequence |
| --- | --- | --- |
| 1 | 28-34 | YDKENGM |
| 2 | 44-50 | DDKNHNK |
| 3 | 99-112 | SDYYPRNSIDTKEY |
| 4 | 159-166 | SPTDKKVG |
| 5 | 178-186 | NWGPYDRDS |
| 6 | 234-244 | MDRKATKQQTN |
| 7 | 251-257 | RVRDDYQ |
| 8 | 268-283 | TNTKDKWTDRSSERYN |
