# Supplementary material for: Integrated immunodominant epitope discovery for dual-purpose rapid and economical diagnostic and immunoprotective applications against MRSA
Source: Front Immunol. 2025 Oct 20;16:1697829. doi: 10.3389/fimmu.2025.1697829 (PMC12580254; doi:10.3389/fimmu.2025.1697829)
Supplement: Supplementary file 11 [file Table5.docx]

Table S5 Predicted immunodominant cytotoxic T lymphocyte (CTL) epitopes of IsdB protein

| Phenotypic classification | Position of the initial amino acid | Sequence | SYFPEITHI（Score） | NetCTL（COMB） |
| --- | --- | --- | --- | --- |
| HLA-A2 * 0201 | 85 | TLYDAIVKV | 30 | 1.4722 |
|  | 35 | MLNGKKYMV | 25 | 1.0172 |
| HLA-A3 * 0301 | 108 | IVDKEAFTK | 29 | 1.0764 |
|  | 76 | IIFPYVEGK | 23 | 1.1834 |
|  | 84 | KTLYDAIVK | 22 | 1.1868 |
|  | 92 | KVHVKTIDY | 20 | 1.3439 |
| HLA-B7 * 0702 | 78 | FPYVEGKTL | 21 | 1.5155 |
| H2-Db | 14 | ESVENNESM | 28 |  |
|  | 68 | DAKNNTRTI | 24 |  |
|  | 15 | SVENNESMM | 20 |  |
| H2-Kk | 57 | VEGQRVRTI | 26 |  |
|  | 3 | TDLQDTKYV | 21 |  |
|  | 23 | MDAFVKHPI | 20 |  |
| H2-Ld | 78 | FPYVEGKTL | 21 |  |
