# Supplementary material for: Integrated immunodominant epitope discovery for dual-purpose rapid and economical diagnostic and immunoprotective applications against MRSA
Source: Front Immunol. 2025 Oct 20;16:1697829. doi: 10.3389/fimmu.2025.1697829 (PMC12580254; doi:10.3389/fimmu.2025.1697829)
Supplement: Supplementary file 12 [file Table6.docx]

Table S6 Predicted immunodominant Helper T cell epitopes of IsdB protein

| Phenotypic classification | Position of the initial amino acid | Sequence | SYFPEITHI（Score） | NetMHC Ⅱ pan 4.0（Rank） |
| --- | --- | --- | --- | --- |
| HLA-DRB1*0301 | 62 | VRTISKDAKNNTRTI | 28 | 0.25 |
|  | 95 | VKTIDYDGQYHVRIV | 27 | 0.43 |
|  | 103 | QYHVRIVDKEAFTKA | 26 | 1.53 |
| HLA-DRB1*0401 | 10 | YVVYESVENNESMMD | 28 | 0.19 |
|  | 7 | DTKYVVYESVENNES | 22 | 0.72 |
|  | 8 | TKYVVYESVENNESM | 20 | 0.53 |
|  | 39 | KKYMVMETTNDDYWK | 20 | 0.72 |
| HLA-DRB1*0701 | 84 | KTLYDAIVKVHVKTI | 24 | 4.46 |
| HLA-DRB1*1101 | 84 | KTLYDAIVKVHVKTI | 26 | 0.94 |
|  | 59 | GQRVRTISKDAKNNT | 20 | 4.84 |
| HLA-DRB1*1501 | 7 | DTKYVVYESVENNES | 24 | 0.86 |
|  | 74 | RTIIFPYVEGKTLYD | 24 | 1.69 |
|  | 72 | NTRTIIFPYVEGKTL | 20 | 4.11 |
| H2-Ad | 130 | GKIGGLIGANVSIGH | 26 |  |
|  | 65 | YRVYSEEGANKSGLA | 24 |  |
|  | 132 | IGGLIGANVSIGHTL | 24 |  |
|  | 214 | NKASSLLSSGFSPDF | 22 |  |
|  | 205 | KAAENFLDPNKASSL | 21 |  |
| H2-Ak | 89 | QLPDNEVAQISDYYP | 24 |  |
|  | 118 | YGFNGNVTGDDSGKI | 22 |  |
|  | 169 | VIFNNMVNQNWGPYD | 22 |  |
|  | 1 | ADSDINIKTGTTDIG | 20 |  |
| H2-Ed | 40 | YSFIDDKNHNKKILV | 28 | 4.29 |
|  | 271 | KDKWTDRSSERYKID | 24 | 3.24 |
|  | 226 | PDFATVITMDRKATK | 20 | 3.31 |
| H2-Ek | 242 | QTNIDVIYERVRDDY | 28 | 2.79 |
|  | 225 | SPDFATVITMDRKAT | 20 | 4.68 |
| H2-Au | 63 | GQYRVYSEEGANKSG |  | 4.74 |
|  | 130 | GKIGGLIGANVSIGH |  | 3 |
|  | 131 | KIGGLIGANVSIGHT |  | 1.76 |
|  | 132 | IGGLIGANVSIGHTL |  | 2.75 |
|  | 133 | GGLIGANVSIGHTLK |  | 4.83 |
|  | 201 | NGSMKAAENFLDPNK |  | 4.22 |
