# Supplementary material for: Integrated immunodominant epitope discovery for dual-purpose rapid and economical diagnostic and immunoprotective applications against MRSA
Source: Front Immunol. 2025 Oct 20;16:1697829. doi: 10.3389/fimmu.2025.1697829 (PMC12580254; doi:10.3389/fimmu.2025.1697829)
Supplement: Supplementary file 13 [file Table7.docx]

Table S7 Predicted immunodominant B cell epitopes of IsdB protein

| No. | Amino acid position | Sequence |
| --- | --- | --- |
| 1 | 1-8 | KMTDLQDT |
| 2 | 15-21 | SVENNES |
| 3 | 46-52 | TTNDDYW |
| 4 | 68-73 | DAKNNT |

Table S8 Predicted immunodominant cytotoxic T lymphocyte (CTL) epitopes of LukG protein

| Phenotypic classification | Position of the initial amino acid | Sequence | SYFPEITHI（Score） | NetCTL（COMB） |
| --- | --- | --- | --- | --- |
| HLA-A2 * 0201 | 286 | ALYEVDWKT | 21 | 0.8702 |
|  | 19 | KMYTRTATT | 20 | 0.937 |
| HLA-A3 * 0301 | 301 | KVLNDNEKK | 26 | 0.9842 |
|  | 156 | LLDQSTSHK | 23 | 1.3516 |
|  | 42 | FLTEPNYDK | 21 | 0.9205 |
|  | 231 | FLAVMSHDK | 21 | 1.1176 |
|  | 293 | KTHNVKFVK | 19 | 1.5115 |
| HLA-B7 * 0702 | 80 | WPGSYSVSI | 19 | 1.3884 |
| H2-Db | 224 | SEGFNPEFL | 26 |  |
|  | 102 | FAPKNQDES | 23 |  |
|  | 94 | NNNTNVTDF | 21 |  |
| H2-Kd | 83 | SYSVSIQNV | 26 |  |
|  | 47 | NYDKETVFI | 20 |  |
|  | 53 | VFIKAKGTI | 20 |  |
|  | 269 | GYWSGENHV | 20 |  |
| H2-Kk | 138 | KESNYSETI | 22 |  |
|  | 190 | SDNRTKSEI | 20 |  |
| H2-Ld | 219 | MPVTVSEGF | 21 |  |
|  | 199 | FSLTRNGNL | 20 |  |

Table S9 Predicted immunodominant Helper T cell epitopes of LukG protein

| Phenotypic classification | Position of the initial amino acid | | Sequence | SYFPEITHI（Score） | NetMHC Ⅱ pan 4.0（Rank） |
| --- | --- | --- | --- | --- | --- |
| HLA-DRB1*0101 | 52 | TVFIKAKGTIGSGLR | | 26 | 1.57 |
|  | 228 | NPEFLAVMSHDKKDK | | 22 | 3.84 |
|  | 51 | ETVFIKAKGTIGSGL | | 21 | 0.37 |
|  | 122 | GGDFSINRGGLTGNI | | 21 | 3.97 |
|  | 150 | QPSYRTLLDQSTSHK | | 20 | 2.37 |
|  | 49 | DKETVFIKAKGTIGS | | 19 | 0.89 |
|  | 50 | KETVFIKAKGTIGSG | | 19 | 0.32 |
| HLA-DRB1*0301 | 232 | LAVMSHDKKDKGKSQ | | 28 | 1.81 |
|  | 11 | EKNLDGDTKMYTRTA | | 26 | 0.45 |
|  | 255 | MDEFKIDWNRHGFWG | | 25 | 3.39 |
|  | 183 | TRQLTNDSDNRTKSE | | 23 | 2.03 |
| HLA-DRB1*0401 | 139 | ESNYSETISYQQPSY | | 22 | 2.77 |
|  | 150 | QPSYRTLLDQSTSHK | | 22 | 1.09 |
|  | 255 | MDEFKIDWNRHGFWG | | 22 | 0.56 |
|  | 85 | SVSIQNVDDNNNTNV | | 20 | 3.12 |
|  | 133 | TGNITKESNYSETIS | | 20 | 5.01 |
|  | 183 | TRQLTNDSDNRTKSE | | 20 | 0.36 |
|  | 252 | KRSMDEFKIDWNRHG | | 20 | 3.48 |
| HLA-DRB1*0701 | 196 | SEIFSLTRNGNLWAK | | 32 | 0.52 |
|  | 18 | TKMYTRTATTSDSQK | | 26 | 1.34 |
|  | 139 | ESNYSETISYQQPSY | | 26 | 0.92 |
|  | 3 | NSEIKQVSEKNLDGD | | 22 | 2.28 |
|  | 137 | TKESNYSETISYQQP | | 20 | 1.61 |
|  | 288 | YEVDWKTHNVKFVKV | | 20 | 4.81 |
|  | 228 | NPEFLAVMSHDKKDK | | 18 | 2.45 |
| HLA-DRB1*1101 | 112 | EVKYTYGYKTGGDFS | | 26 | 1.17 |
|  | 71 | NGYWNSTLRWPGSYS | | 24 | 1.06 |
|  | 229 | PEFLAVMSHDKKDKG | | 22 | 5.40 |
|  | 195 | KSEIFSLTRNGNLWA | | 21 | 0.82 |
|  | 165 | GVGWKVEAHLINNMG | | 20 | 4.02 |
|  | 210 | KDNFTPKDKMPVTVS | | 20 | 1.57 |
|  | 244 | KSQFVVHYKRSMDEF | | 20 | 2.29 |
|  | 285 | SALYEVDWKTHNVKF | | 19 | 4.47 |
| HLA-DRB1*1501 | 110 | SREVKYTYGYKTGGD | | 18 | 2.15 |
| H2-Ad | 164 | KGVGWKVEAHLINNM | | 24 |  |
|  | 17 | DTKMYTRTATTSDSQ | | 22 |  |
|  | 19 | KMYTRTATTSDSQKN | | 22 |  |
|  | 49 | DKETVFIKAKGTIGS | | 22 |  |
|  | 215 | PKDKMPVTVSEGFNP | | 22 |  |
|  | 278 | DKKEEKLSALYEVDW | | 21 |  |
|  | 80 | WPGSYSVSIQNVDDN | | 20 |  |
|  | 107 | QDESREVKYTYGYKT | | 20 |  |
|  | 149 | QQPSYRTLLDQSTSH | | 20 |  |
|  | 243 | GKSQFVVHYKRSMDE | | 20 |  |
|  | 291 | DWKTHNVKFVKVLND | | 20 |  |
| H2-Ak | 92 | DDNNNTNVTDFAPKN | | 22 |  |
|  | 151 | PSYRTLLDQSTSHKG | | 22 |  |
|  | 189 | DSDNRTKSEIFSLTR | | 22 |  |
|  | 222 | TVSEGFNPEFLAVMS | | 22 |  |
|  | 14 | LDGDTKMYTRTATTS | | 20 |  |
|  | 72 | GYWNSTLRWPGSYSV | | 20 |  |
|  | 89 | QNVDDNNNTNVTDFA | | 20 |  |
| H2-Au | 16 | GDTKMYTRTATTSDS | |  | 3.54 |
|  | 17 | DTKMYTRTATTSDSQ | |  | 2.15 |
|  | 18 | TKMYTRTATTSDSQK | |  | 1.57 |
|  | 19 | KMYTRTATTSDSQKN | |  | 2.42 |
|  | 110 | SREVKYTYGYKTGGD | |  | 3.87 |
|  | 167 | GWKVEAHLINNMGHD | |  | 4.83 |
|  | 267 | FWGYWSGENHVDKKE | |  | 4.85 |
|  | 294 | THNVKFVKVLNDNEK | |  | 3.76 |
| H2-Ed | 229 | PEFLAVMSHDKKDKG | | 22 | 0.11 |
|  | 45 | EPNYDKETVFIKAKG | | 20 | 0.74 |
| H2-Ek | 228 | NPEFLAVMSHDKKDK | | 20 | 0.35 |

Table S10 Predicted immunodominant B cell epitopes of LukG protein

| No. | Amino acid position | Sequence |
| --- | --- | --- |
| 1 | 45-51 | PNYDKET |
| 2 | 92-98 | DNNNTNV |
| 3 | 102-114 | APKNQDESREVKY |
| 4 | 137-143 | QESNYSE |
| 5 | 181-196 | HTRQLTNDSDNRTKSE |
| 6 | 237-244 | DKKDKGKS |
| 7 | 276-283 | VDKKEEKL |
| 8 | 305-309 | LNDNE |

Table S11 Predicted immunodominant cytotoxic T lymphocyte (CTL) epitopes of MntC protein

| Phenotypic classification | Position of the initial amino acid | Sequence | SYFPEITHI（Score） | NetCTL（COMB） |
| --- | --- | --- | --- | --- |
| HLA-A2 * 0201 | 120 | SLDNGIKYV | 27 | 1.2106 |
|  | 17 | ILYDMAKNV | 23 | 0.9554 |
|  | 54 | KLTDADVIL | 23 | 1.2292 |
|  | 194 | ITPGYIWEI | 23 | 1.0640 |
|  | 272 | MMKSNIETV | 23 | 1.0807 |
|  | 212 | QMRQAIEFV | 22 | 0.8512 |
| HLA-A3 * 0301 | 228 | LLVETSVDK | 25 | 0.8801 |
|  | 159 | KLNNDSKDK | 24 | 1.1039 |
|  | 178 | MITSEGAFK | 24 | 1.2886 |
|  | 253 | EVYTDSIGK | 24 | 0.9599 |
|  | 102 | YLNGEEGNK | 23 | 0.7672 |
|  | 198 | YIWEINTEK | 20 | 0.9572 |
|  | 214 | RQAIEFVKK | 20 | 1.1640 |
| HLA-B7 * 0702 | 171 | IPKEQRAMI | 19 | 1.3868 |
|  | 209 | TPEQMRQAI | 18 | 1.3915 |
| H2-Db | 60 | VILYNGLNL | 22 | 60 |
|  | 272 | MMKSNIETV | 22 | 272 |
| H2-Kd | 152 | KYIAQLEKL | 24 | 152 |
|  | 186 | KYFSKQYGI | 24 | 186 |
|  | 44 | EYEVKPKDI | 23 | 44 |
|  | 269 | YYKMMKSNI | 23 | 269 |
|  | 191 | QYGITPGYI | 21 | 191 |
|  | 226 | KHLLVETSV | 20 | 226 |
| H2-Kk | 146 | YEKQGNKYI | 22 | 146 |
|  | 27 | GDNVDIHSI | 21 | 27 |
| H2-Ld | 180 | TSEGAFKYF | 21 | 180 |

Table S12 Predicted immunodominant Helper T cell epitopes of MntC protein

| Phenotypic classification | Position of the initial amino acid | | Sequence | SYFPEITHI（Score） | NetMHC Ⅱ pan 4.0（Rank） |
| --- | --- | --- | --- | --- | --- |
| HLA-DRB1*0101 | 6 | NGKLKVVTTNSILYD | | 29 | 4.70 |
|  | 143 | KADYEKQGNKYIAQL | | 28 | 2.39 |
|  | 49 | PKDIKKLTDADVILY | | 27 | 4.16 |
|  | 266 | GDSYYKMMKSNIETV | | 26 | 1.58 |
|  | 14 | TNSILYDMAKNVGGD | | 25 | 1.20 |
|  | 267 | DSYYKMMKSNIETVH | | 24 | 1.03 |
|  | 29 | NVDIHSIVPVGQDPH | | 22 | 4.43 |
| HLA-DRB1*0301 | 157 | LEKLNNDSKDKFNDI | | 36 | 0.17 |
|  | 91 | VIAVSKDVKPIYLNG | | 34 | 0.22 |
|  | 116 | HAWLSLDNGIKYVKT | | 30 | 0.61 |
|  | 133 | QTFIDNDKKHKADYE | | 28 | 0.27 |
|  | 214 | RQAIEFVKKHKLKHL | | 26 | 3.77 |
|  | 14 | TNSILYDMAKNVGGD | | 23 | 0.82 |
|  | 176 | RAMITSEGAFKYFSK | | 22 | 2.33 |
|  | 251 | FGEVYTDSIGKEGTK | | 22 | 2.78 |
|  | 35 | IVPVGQDPHEYEVKP | | 21 | 1.90 |
|  | 8 | KLKVVTTNSILYDMA | | 20 | 4.79 |
| HLA-DRB1*0401 | 195 | TPGYIWEINTEKQGT | | 28 | 0.90 |
|  | 6 | NGKLKVVTTNSILYD | | 26 | 2.09 |
|  | 196 | PGYIWEINTEKQGTP | | 26 | 0.72 |
|  | 16 | SILYDMAKNVGGDNV | | 22 | 0.26 |
|  | 14 | TNSILYDMAKNVGGD | | 20 | 0.86 |
|  | 29 | NVDIHSIVPVGQDPH | | 20 | 2.82 |
| HLA-DRB1*0701 | 8 | KLKVVTTNSILYDMA | | 30 | 2.34 |
|  | 6 | NGKLKVVTTNSILYD | | 24 | 0.31 |
|  | 124 | GIKYVKTIQQTFIDN | | 24 | 1.22 |
|  | 143 | KADYEKQGNKYIAQL | | 24 | 4.94 |
|  | 189 | SKQYGITPGYIWEIN | | 24 | 3.10 |
|  | 7 | GKLKVVTTNSILYDM | | 22 | 0.98 |
|  | 125 | IKYVKTIQQTFIDND | | 22 | 4.39 |
| HLA-DRB1*1101 | 15 | NSILYDMAKNVGGDN | | 27 | 0.53 |
|  | 165 | KDKFNDIPKEQRAMI | | 24 | 2.83 |
|  | 182 | EGAFKYFSKQYGITP | | 24 | 0.08 |
|  | 266 | GDSYYKMMKSNIETV | | 24 | 1.31 |
|  | 88 | DKKVIAVSKDVKPIY | | 21 | 3.83 |
|  | 133 | QTFIDNDKKHKADYE | | 21 | 2.93 |
|  | 214 | RQAIEFVKKHKLKHL | | 21 | 1.02 |
|  | 42 | PHEYEVKPKDIKKLT | | 20 | 3.75 |
|  | 151 | NKYIAQLEKLNNDSK | | 20 | 0.60 |
| HLA-DRB1*1501 | 57 | DADVILYNGLNLETG | | 34 | 0.05 |
|  | 59 | DVILYNGLNLETGNG | | 24 | 2.03 |
|  | 122 | DNGIKYVKTIQQTFI | | 24 | 1.75 |
| H2-Ad | 222 | KHKLKHLLVETSVDK | | 28 |  |
|  | 60 | VILYNGLNLETGNGW | | 24 |  |
|  | 230 | VETSVDKKAMESLSE | | 24 |  |
|  | 54 | KLTDADVILYNGLNL | | 23 |  |
|  | 11 | VVTTNSILYDMAKNV | | 22 |  |
|  | 113 | QDPHAWLSLDNGIKY | | 21 |  |
|  | 92 | IAVSKDVKPIYLNGE | | 20 |  |
| H2-Ak | 232 | TSVDKKAMESLSEET | | 26 |  |
|  | 166 | DKFNDIPKEQRAMIT | | 22 |  |
|  | 61 | ILYNGLNLETGNGWF | | 20 |  |
|  | 148 | KQGNKYIAQLEKLNN | | 20 |  |
| H2-Au | 15 | NSILYDMAKNVGGDN | |  |  |
|  | 27 | GDNVDIHSIVPVGQD | |  |  |
|  | 28 | DNVDIHSIVPVGQDP | |  |  |
|  | 29 | NVDIHSIVPVGQDPH | |  |  |
|  | 30 | VDIHSIVPVGQDPHE | |  |  |
|  | 251 | FGEVYTDSIGKEGTK | |  |  |
|  | 252 | GEVYTDSIGKEGTKG | |  |  |
|  | 267 | DSYYKMMKSNIETVH | |  |  |
| H2-Ed | 42 | PHEYEVKPKDIKKLT | | 24 | 0.91 |
|  | 216 | AIEFVKKHKLKHLLV | | 24 | 2.59 |
|  | 165 | KDKFNDIPKEQRAMI | | 22 | 0.84 |
|  | 130 | TIQQTFIDNDKKHKA | | 20 | 3.28 |
| H2-Ek | 88 | DKKVIAVSKDVKPIY | | 24 | 0.23 |

Table S13 Predicted immunodominant B cell epitopes of MntC protein

| No. | Amino acid position | Sequence |
| --- | --- | --- |
| 1 | 1-7 | SSDKSNG |
| 2 | 41-56 | DPHEYEVKPKDIKKLP |
| 3 | 107-115 | EGNKDKQDPH |
| 4 | 138-151 | NDKKHKADYEKQGN |
| 5 | 160-175 | LNNDSKDKFNDIPKEN |
| 6 | 203-213 | NTEKQGTPEQM |
| 7 | 262-274 | EGTKGDSYYKMMK |

Table S14 Predicted immunodominant cytotoxic T lymphocyte (CTL) epitopes of SEB protein

| Phenotypic classification | Position of the initial amino acid | Sequence | SYFPEITHI（Score） | NetCTL（COMB） |
| --- | --- | --- | --- | --- |
| HLA-A2 * 0201 | 20 | GLMENMKVL | 23 | 0.9249 |
|  | 46 | RYFDLIYSI | 18 | 0.8425 |
|  | 216 | MMYNDNKMV | 18 | 1.1287 |
| HLA-A3 * 0301 | 163 | YLTRHYLVK | 30 | 1.3407 |
|  | 50 | LIYSIKDTK | 26 | 1.1471 |
|  | 230 | KIEVYLTTK | 26 | 1.1083 |
|  | 232 | EVYLTTKKK | 25 | 1.0341 |
|  | 155 | KVTAQELDY | 24 | 1.0422 |
|  | 90 | AYYQCAFSK | 22 | 1.1045 |
|  | 166 | RHYLVKNKK | 20 | 0.7500 |
|  | 214 | YLMMYNDNK | 20 | 0.7779 |
| H2-Db | 34 | VSAINVKSI | 24 |  |
|  | 189 | KFIENENSF | 23 |  |
|  | 20 | GLMENMKVL | 22 |  |
|  | 139 | EDGKNLLSF | 22 |  |
|  | 215 | LMMYNDNKM | 22 |  |
|  | 57 | TKLGNYDNV | 21 |  |
|  | 119 | VTEHNGNQL | 20 |  |
| H2-Kd | 46 | RYFDLIYSI | 26 |  |
|  | 167 | HYLVKNKKL | 26 |  |
|  | 51 | IYSIKDTKL | 24 |  |
|  | 162 | DYLTRHYLV | 24 |  |
|  | 77 | KYKDKYVDV | 22 |  |
|  | 129 | KYRSITVRV | 22 |  |
|  | 137 | VFEDGKNLL | 20 |  |
| H2-Kk | 183 | YETGYIKFI | 23 |  |
|  | 29 | YDDNHVSAI | 20 |  |
| H2-Ld | 4 | QPDPKPDEL | 22 |  |
|  | 40 | KSIDQFRYF | 20 |  |

Table S15 Predicted immunodominant Helper T cell epitopes of SEB protein

| Phenotypic classification | Position of the initial amino acid | Sequence | SYFPEITHI（Score） | NetMHC Ⅱ pan 4.0（Rank） |
| --- | --- | --- | --- | --- |
| HLA-DRB1*0101 | 79 | KDKYVDVFGANAYYQ | 33 | 0.51 |
|  | 127 | LDKYRSITVRVFEDG | 27 | 4.49 |
|  | 196 | SFWYDMMPAPGDKFD | 27 | 3.83 |
|  | 195 | NSFWYDMMPAPGDKF | 22 | 2.71 |
| HLA-DRB1*0301 | 134 | TVRVFEDGKNLLSFD | 38 | 0.11 |
|  | 24 | NMKVLYDDNHVSAIN | 28 | 0.77 |
|  | 18 | FTGLMENMKVLYDDN | 27 | 3.46 |
|  | 142 | KNLLSFDVQTNKKKV | 27 | 0.83 |
|  | 214 | YLMMYNDNKMVDSKD | 26 | 1.58 |
|  | 25 | MKVLYDDNHVSAINV | 21 | 1.20 |
| HLA-DRB1*0401 | 24 | NMKVLYDDNHVSAIN | 26 | 0.54 |
|  | 25 | MKVLYDDNHVSAINV | 26 | 2.07 |
|  | 185 | TGYIKFIENENSFWY | 26 | 0.74 |
|  | 79 | KDKYVDVFGANAYYQ | 22 | 2.91 |
|  | 135 | VRVFEDGKNLLSFDV | 22 | 3.16 |
|  | 184 | ETGYIKFIENENSFW | 22 | 0.87 |
|  | 211 | QSKYLMMYNDNKMVD | 22 | 3.66 |
|  | 48 | FDLIYSIKDTKLGNY | 20 | 4.76 |
|  | 134 | TVRVFEDGKNLLSFD | 20 | 3.28 |
|  | 141 | GKNLLSFDVQTNKKK | 20 | 4.58 |
|  | 172 | NKKLYEFNNSPYETG | 20 | 1.66 |
| HLA-DRB1*0701 | 124 | GNQLDKYRSITVRVF | 24 | 4.97 |
|  | 31 | DNHVSAINVKSIDQF | 22 | 4.62 |
|  | 126 | QLDKYRSITVRVFED | 22 | 1.03 |
|  | 127 | LDKYRSITVRVFEDG | 22 | 0.77 |
| HLA-DRB1*1101 | 64 | NVRVEFKNKDLADKY | 21 | 2.79 |
|  | 9 | PDELHKSSKFTGLME | 20 | 2.55 |
|  | 158 | AQELDYLTRHYLVKN | 20 | 3.01 |
|  | 165 | TRHYLVKNKKLYEFN | 20 | 2.29 |
|  | 127 | LDKYRSITVRVFEDG | 18 | 4.82 |
| HLA-DRB1*1501 | 56 | DTKLGNYDNVRVEFK | 34 | 0.05 |
|  | 124 | GNQLDKYRSITVRVF | 34 | 0.15 |
|  | 80 | DKYVDVFGANAYYQC | 24 | 3.96 |
|  | 141 | GKNLLSFDVQTNKKK | 24 | 4.92 |
|  | 172 | NKKLYEFNNSPYETG | 24 | 0.73 |
|  | 212 | SKYLMMYNDNKMVDS | 24 | 0.32 |
|  | 226 | SKDVKIEVYLTTKKK | 20 | 1.53 |
| H2-Ad | 77 | KYKDKYVDVFGANAY | 29 |  |
|  | 28 | LYDDNHVSAINVKSI | 27 |  |
|  | 150 | QTNKKKVTAQELDYL | 24 |  |
|  | 31 | DNHVSAINVKSIDQF | 23 |  |
|  | 129 | KYRSITVRVFEDGKN | 22 |  |
|  | 79 | KDKYVDVFGANAYYQ | 21 |  |
|  | 113 | TCMYGGVTEHNGNQL | 20 |  |
|  | 127 | LDKYRSITVRVFEDG | 20 |  |
| H2-Ak | 77 | KYKDKYVDVFGANAY | 24 |  |
|  | 169 | LVKNKKLYEFNNSPY | 20 |  |
| H2-Au | 29 | YDDNHVSAINVKSID |  | 3.4 |
|  | 30 | DDNHVSAINVKSIDQ |  | 1.79 |
|  | 31 | DNHVSAINVKSIDQF |  | 3.84 |
|  | 112 | KTCMYGGVTEHNGNQ |  | 3.67 |
|  | 126 | QLDKYRSITVRVFED |  | 2.34 |
|  | 127 | LDKYRSITVRVFEDG |  | 1.71 |
|  | 128 | DKYRSITVRVFEDGK |  | 2.79 |
| H2-Ed | 66 | RVEFKNKDLADKYKD | 26 | 0.29 |
|  | 144 | LLSFDVQTNKKKVTA | 26 | 0.65 |
|  | 100 | TNDINSHQTDKRKTC | 22 | 2.44 |
|  | 59 | LGNYDNVRVEFKNKD | 20 | 1.72 |
| H2-Ek | 47 | YFDLIYSIKDTKLGN | 24 | 3.34 |
|  | 185 | TGYIKFIENENSFWY | 20 | 2.49 |

Table S16 Predicted immunodominant B cell epitopes of SEB protein

| No. | Amino acid position | Sequence |
| --- | --- | --- |
| 1 | 1-16 | MESQPDPKPDELHKSE |
| 2 | 68-81 | EFKNKDLADKYKDK |
| 3 | 97-112 | SKKTNDINSHQTDKRK |
| 4 | 122-131 | HNGNQLDKYR |
| 5 | 150-157 | QTNKKKVT |
| 6 | 170-185 | VKNKKLYEFNNSPYEK |
| 7 | 208-214 | KFDQSKY |
| 8 | 218-229 | YNDNKMVDSKDV |
